# Targeted Disruption of Ing2 Results in Defective Spermatogenesis and Development of Soft-Tissue Sarcomas

**DOI:** 10.1371/journal.pone.0015541

**Published:** 2010-11-19

**Authors:** Motonobu Saito, Kensuke Kumamoto, Ana I. Robles, Izumi Horikawa, Bungo Furusato, Shu Okamura, Akiteru Goto, Taro Yamashita, Makoto Nagashima, Tin-Lap Lee, Vanessa J. Baxendale, Owen M. Rennert, Seiichi Takenoshita, Jun Yokota, Isabell A. Sesterhenn, Glenwood E. Trivers, S. Perwez Hussain, Curtis C. Harris

**Affiliations:** 1 Laboratory of Human Carcinogenesis, Center for Cancer Research, National Cancer Institute, National Institutes of Health, Bethesda, Maryland, United States of America; 2 Department of Organ Regulatory Surgery, Fukushima Medical University School of Medicine, Fukushima, Japan; 3 Department of Genitourinary Pathology, Armed Forces Institute of Pathology, Washington, D.C., United States of America; 4 Laboratory of Clinical and Developmental Genomics, Program in Reproductive and Adult Endocrinology, National Institute of Child Health and Human Development, National Institutes of Health, Bethesda, Maryland, United States of America; 5 Biology Division, National Cancer Center Research Institute, Tokyo, Japan; Seattle Children's Research Institute, United States of America

## Abstract

ING2 (inhibitor of growth family, member 2) is a member of the plant homeodomain (PHD)-containing ING family of putative tumor suppressors. As part of mSin3A-HDAC corepressor complexes, ING2 binds to tri-methylated lysine 4 of histone H3 (H3K4me3) to regulate chromatin modification and gene expression. ING2 also functionally interacts with the tumor suppressor protein p53 to regulate cellular senescence, apoptosis and DNA damage response *in vitro*, and is thus expected to modulate carcinogenesis and aging. Here we investigate the developmental and physiological functions of Ing2 through targeted germline disruption. Consistent with its abundant expression in mouse and human testes, male mice deficient for *Ing2* showed abnormal spermatogenesis and were infertile. Numbers of mature sperm and sperm motility were significantly reduced in *Ing2* ^−/−^ mice (∼2% of wild type, *P*<0.0001 and ∼10% of wild type, *P*<0.0001, respectively). Their testes showed degeneration of seminiferous tubules, meiotic arrest before pachytene stage with incomplete meiotic recombination, induction of p53, and enhanced apoptosis. This phenotype was only partially abrogated by concomitant loss of p53 in the germline. The arrested spermatocytes in *Ing2* ^−/−^ testes were characterized by lack of specific HDAC1 accumulation and deregulated chromatin acetylation. The role of Ing2 in germ cell maturation may extend to human ING2 as well. Using publicly available gene expression datasets, low expression of ING2 was found in teratozoospermic sperm (>3-fold reduction) and in testes from patients with defective spermatogenesis (>7-fold reduction in Sertoli-cell only Syndrome). This study establishes ING2 as a novel regulator of spermatogenesis functioning through both p53- and chromatin-mediated mechanisms, suggests that an HDAC1/ING2/H3K4me3-regulated, stage-specific coordination of chromatin modifications is essential to normal spermatogenesis, and provides an animal model to study idiopathic and iatrogenic infertility in men. In addition, a bona fide tumor suppressive role of Ing2 is demonstrated by increased incidence of soft-tissue sarcomas in *Ing2^−^* ^/*−*^ mice.

## Introduction

ING2 (inhibitor of growth family, member 2) plays pivotal roles in the regulation of cellular senescence, apoptosis, DNA damage repair, gene transcription and chromatin modification [1], [2]. Our previous *in vitro* studies on cellular senescence suggested that ING2 functionally interplays with the p53 tumor suppressor protein in two different manners: endogenous ING2 inhibits senescence and the transcriptional repression of *ING2* by p53 abrogates this inhibition [3]; and overexpressed ING2 enhances p53 acetylation and stability to induce senescence [4], [5]. ING2, as a subunit of the mSin3A-HDAC1 (histone deacetylase 1) complex, specifically binds to tri-methylated lysine 4 of histone H3 (H3K4me3) via its plant homeodomain (PHD) finger and regulates gene expression through chromatin modifications in response to DNA damage [6], [7]. Although these findings imply that ING2 may contribute *in vivo* to p53-regulated processes, as well as developmental and homeostatic processes involving chromatin regulation, the *in vivo* physiological roles of ING2 have not been experimentally examined.

Spermatogenesis, a series of spermatogenic cell differentiation steps from spermatogonia to mature spermatozoa in the testes, is a process tightly regulated by chromatin modifications [8], [9]. Enzymes that modify histone methylation, including Meisetz (an H3K4 tri-methyltransferase) [10], G9a [a mono- and di-methyltransferase on lysine 9 of histone H3 (H3K9)] [11], and Suv39h1 and Suv39h2 (H3K9 tri-methyltransferases) [12], are essential for normal germ cell development in mice. The stage-specific acetylation profiles of several lysine residues on core histones, i.e., acetylation in spermatogonia and deacetylation during differentiation from leptotene to pachytene stages, are also critical to normal spermatogenesis in mice [8], [13]. Consistently, HDAC inhibitors impaired male fertility in mice through loss of pachytene spermatocytes and increased apoptosis [14], [15], [16]. Such dynamic regulation of chromatin modifications during spermatogenesis is not limited to mice: the H3K4 methylation profiles during spermatogenesis were very similar between mice and non-human primates [17]. Misregulated histone acetylation is associated with defective spermatogenesis in humans [18], suggesting that chromatin-mediated regulation is a conserved mechanism from rodents to humans. However, no specific gene defect has been identified in humans as responsible for spermatogenic defect due to aberrant chromatin regulation [19].

In this study, our generation and characterization of *Ing2*-deficient mice reveal that ING2 plays an essential *in vivo* role in mammalian spermatogenesis, which is attributed to its functional interaction with p53 and chromatin regulation. The relevance in humans is underscored by bioinformatics analysis revealing low ING2 expression in men with infertility and defective spermatogenesis. In addition, loss of Ing2 resulted in high incidence of soft-tissue sarcomas, particularly histiocytic sarcomas, demonstrating, for the first time, a tumor suppressor role for Ing2.

## Results

### ING2 is expressed abundantly in mouse and human testes

**Figure 1:**
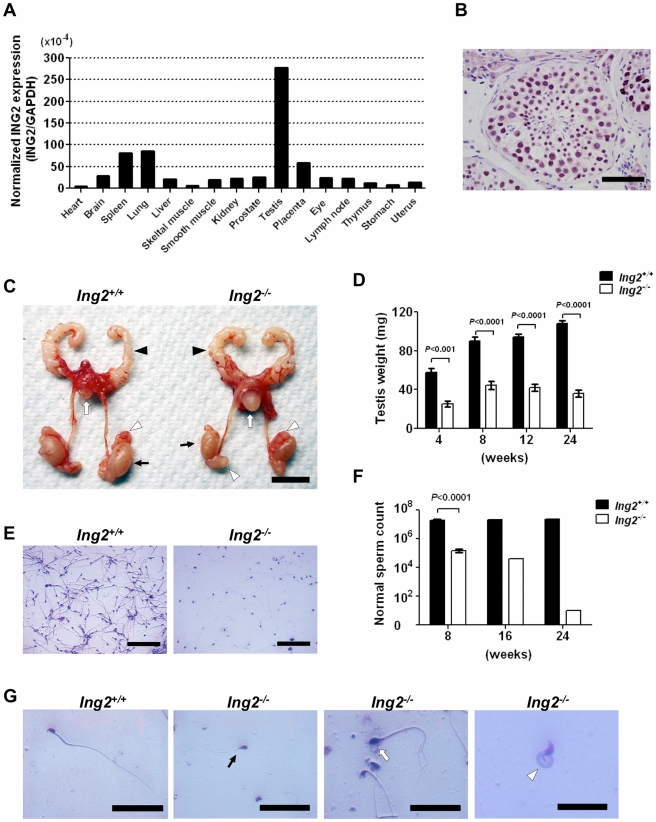
Testicular atrophy and semen abnormalities in *Ing2^−/−^* mice. (A) ING2 is abundant in testes. qRT-PCR analysis of ING2 expression in mouse tissues. ING2 expression levels (normalized to GAPDH) are shown on a scale of 10^−4^. (B) Immunohistochemical (IHC) staining of ING2 protein in a normal human testis section. Positive nuclear staining is evident in the seminiferous tubule. Scale bar is 100 µm. (C) Morphology of reproductive organs from 8-week-old *Ing2^+/+^* and *Ing2^−/−^* male mice. The seminal vesicles (black arrowheads), bladder (white arrows), epididymis (white arrowheads), testes (black arrows) are demonstrated. Scale bar, 1 cm. (D) Testis weight during postnatal development of *Ing2^+/+^* and *Ing2^−/−^* mice. Student's *t* test, n = 5 per group. Error bars are s.e.m. (E) Semen in *Ing2^+/+^* and *Ing2^−/−^* mice. Scale bars, 100 µm. (F) Numbers of normal spermatozoa in semen during postnatal development of *Ing2^+/+^* and *Ing2^−/−^* mice. Data are mean from n = 3 (8 weeks) or n = 2 (16 and 24 weeks). Error bars are s.e.m. (G) Normal and abnormal spermatozoa from *Ing2^+/+^* and *Ing2^−/−^* mice, respectively. The leftmost panel (*Ing2^+/+^*) shows mature normal spermatozoon with a characteristic, hook-shaped nucleus, straight mitochondrial sheath and straight tail. The right three panels (*Ing2^−/−^*) show morphologically abnormal, immature spermatozoa, including ones with a round head and a short tail (black arrow), a large head and multiple tails (white arrow), and a tail coiled (white arrowhead). Scale bars are 50 µm in the left three panels and 25 µm in the rightmost panel.

Quantitative RT-PCR (qRT-PCR) analysis of Ing2 mRNA expression in normal mouse tissues demonstrated a tissue-specific expression pattern, with testis showing the highest level of Ing2 (Fig. 1A), as has been reported in humans [20]. Immunohistochemical (IHC) staining of human testis sections showed that the cells in seminiferous tubules expressed higher levels of ING2 protein than the interstitial cells (Fig. 1B, Fig. S1). These data indicate that germ cells are the major source of ING2 expression in mouse and human testes.

### Generation of *Ing2*-deficient mice

To examine the *in vivo* developmental and physiological roles of ING2, with particular interest to testicular development and function, mouse *Ing2* gene was knocked out using a Cre-loxP recombination system (Fig. S2A). DNA genotyping (Fig. S2B), qRT-PCR analysis (Fig. S2C) and western blot analysis (Fig. S2D) confirmed the generation of mice with wild-type *Ing2* (+/+), heterozygous for *Ing2* knockout (+/−) and homozygous for *Ing2* knockout (−/−). In crosses between heterozygous mice, the occurrences of *Ing2^+/+^*, *Ing2^+/−^* and *Ing2^−/−^* genotypes were 27% (154 out of 570), 56% (319 out of 570) and 17% (97 out of 570), respectively, showing a slight deviation from the expected Mendelian distribution and indicating that Ing2 deficiency may have a mild adverse effect on embryonic or prenatal development.

### *Ing2*-deficient males are infertile

The postnatal growth of *Ing2^−/−^* mice was indistinguishable from that of their *Ing2^+/+^* littermates. *Ing2^−/−^* mice had significantly smaller testes than those of *Ing2^+/+^* mice throughout their life (Fig. 1C,D and Table 1, *P*<0.001), but did not show any gross abnormalities in other organs, including seminal vesicles, epididymides and vasa deferens (Fig. 1C, Table 1). The small-sized testes of *Ing2^−/−^* mice were not due to serum testosterone levels (Table 1).

**Table 1:** Weight of organs, serum testosterone levels, and sperm parameters in *Ing2^+/+^* and *Ing2^−/−^* male mice.

		*Ing2^+/+^*	*Ing2^−/−^*	*P* value^a^
Total body weight (g)^b^		25.9±4.0	23.6±1.4	NS
Organ Weight (mg)b, c	Brain	321±10.8	312±8.6	NS
	Thymus	79.2±30.1	59.6±6.8	NS
	Lung	86.1±6.3	76.1±14.6	NS
	Heart	153±16.9	143±37.7	NS
	Spleen	103±19.8	79.4±7.7	NS
	Kidney	192±27.3	167±12.9	NS
	Liver	1539±432	1353±176	NS
	Stomach	234±32.3	221±77.4	NS
	Intestine, pancreas, mesenterium	2303±248	2027±448	NS
	Seminal vesicle	105±19.4	83.1±11	NS
	Bladder and prostate	132±22.7	108±19.0	NS
	Epididymis	19.5±2.6	17.9±7.1	NS
	Testis	91.8±13.6	45.2±11.2	0.0004
Serum testosterone (ng/dl)^d^	8 wks	88.6±22	135±48	NS
	26 wks	66.4±17	44.8±19	NS
Sperm parameters^e^	Total no. of cells(x10^6^)	44.4±5.8	9.8±1.7	0.005
	No. of normal sperm (x10^6^)	32±7.5	0.7±0.2	<0.0001
	Sperm motility (%)	91.0±4.3	11.4±3.2	<0.0001

a Student's *t* test, NS; Not significant.

b 8-week-old mice were examined. n = 5 per group. Values are means ± SD.

c The wet weights of paired organs were averaged for each mouse, and this single value was used to calculate mean ± SD among same genotype.

d n = 5 per group. Values are means ± SD.

e n = 3 per group. Values are means ± SD.

When 8-week-old *Ing2^+/+^*, *Ing2^+/−^* and *Ing2^−/−^* mice were mated, *Ing2^−/−^* male mice were revealed to be infertile, while *Ing*2^+/*−*^ male and *Ing*2*^−^* ^/*−*^ female mice were fertile. The number of normal sperm found in semen from epididymis and vas deferens in 8-week-old *Ing2^−/−^* males was only approximately 2% of that in age-matched *Ing2^+/+^* control (Fig. 1E, F and Table 1, *P*<0.0001). The low sperm count in *Ing2^−/−^* mice became more severe with aging (Fig. 1F). In *Ing2^−/−^* mice, the sperm motility was also severely impaired (Table 1, *P*<0.0001) and almost all spermatozoa showed abnormal morphologies, such as round heads, short tails, large heads, multiple tails and tail coiling (Fig. 1G). Thus, male infertility in *Ing2^−/−^* mice is attributed to quantitative and qualitative defects in mature spermatozoa.

### *Ing2^−/−^* testes show degeneration of seminiferous tubules

**Figure 2:**
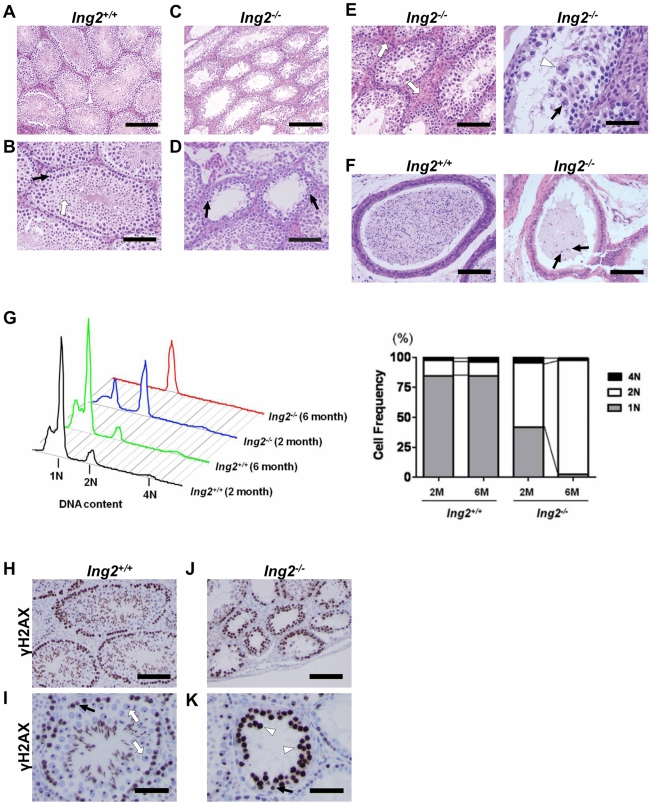
Degeneration of seminiferous tubules and meiotic arrest in *Ing2^−/−^* testes. (A–E) Histological analysis of testis sections from 8-week-old *Ing2^+/+^* (A, B) and *Ing2^−/−^* (C–E) mice by hematoxylin and eosin (H&E) staining. Black arrows in (B) and (D) indicate spermatocytes with large, condenced nuclei. The white arrow in (B) indicates spermatids, which are missing in (D). In (E), Leydig cell hyperplasia (white arrows), apoptotic cell (black arrow) and multinucleated giant cell (white arrowhead) are indicated. Scale bars are 200 µm in (A) and (C), 100 µm in (B), (D) and (E, left), and 50 µm in (E, right). (F) Histological analysis of epididymis from 8-week-old *Ing2^+/+^* and *Ing2^−/−^* mice by H&E staining. Scale bars, 100 µm. Black arrows indicate degenerated round cells in *Ing2^−/−^* epididymis. (G) Flow cytometric analysis of testis cells isolated from 2- and 6-month-old *Ing2^+/+^* and *Ing2^−/−^* mice. The flow cytograms (left) demonstrate three peaks; 1N peak representing round and elongated spermatids, 2N peak representing somatic cells, spermatogonia and secondary spermatocytes, and 4N peak representing primary spermatocytes, including leptotene, zygotene and pachytene stages. The data are shown as percentage of 1N, 2N and 4N cell fractions (right). (H–K) Phosphorylated histone H2AX (γ-H2AX) staining in testes from 8-week-old *Ing2^+/+^* (H, I) and *Ing2^−/−^* (J, K) mice. Normal leptotene and zygotene spermatocytes with positive γ-H2AX staining [black arrows in (I) and (K)] develop into pachytene spermatocytes in *Ing2^+/+^* testes [white arrows in (I)], which have a γ-H2AX focus corresponding to the sex body but are otherwise negative for γ-H2AX staining [21]. In *Ing2^−/−^* testes, γ-H2AX-positive, abnormal spermatocytes accumulate [white arrowheads in (K)] without development into pachytene spermatocytes. Scale bars are 100 µm in (H) and (J), and 50 µm in (I) and (K).

Histological analysis showed normal germ cell development in seminiferous tubules in *Ing2^+/+^* testes (Fig. 2A,B). In contrast, *Ing2^−/−^* testes exhibited seminiferous tubule degeneration, germ cell under-population (Fig. 2C,D) with Leydig cell hyperplasia, apoptotic cells and multinucleated giant cells (Fig. 2E). In *Ing2^−/−^* seminiferous tubules, spermatogonia are present in low numbers and in some tubules various developmental stages can be seen up to round spermatid. The major cell stage observed in these tubules is spermatocyte with large dense nuclei (Fig. 2D, 2B, black arrows). However, postmeiotic cell types such as round and elongated spermatids (Fig. 2B, white allow) were scarcely observed in *Ing2^−/−^* tubules (Fig. 2D), suggesting a spermatogenesis arrest at meiotic phase in most tubules. Observation of the epididymal wall also suggests that the cellular organization of this structure is also compromised in *Ing2^−/−^* mice. While mature spermatozoa were present in the epididymides of *Ing2^+/+^* mice, hypospermia was observed in *Ing2^−/−^* (Fig. 2F).

*Ing2^+/+^* and *Ing2^−/−^* testes were histologically examined throughout the mouse lifespan (Fig. S3). While *Ing2^+/+^* seminiferous tubules developed normally and became completely populated with all stages of germ cells by 6 to 8 weeks, most *Ing2^−/−^* tubules remained devoid of germ cells and/or disorganized at the same age. Analysis at 6 through 24 months of age reveals that *Ing2^+/+^* tubules maintained normal germ cell development. *Ing2^−/−^* tubules, however, underwent progressive degeneration and germ cell depletion, eventually showing a Sertoli-cell-only pathology at 24 months. When the epididymis was examined at 24 months, mature spermatozoa were present in *Ing2^+/+^*, while the *Ing2^−/−^* epididymis displayed an aspermic phenotype.

### Meiotic arrest and impaired meiotic recombination in *Ing2^−/−^* testes

DNA contents were analyzed in cells from 2- and 6-month-old *Ing2^+/+^* and *Ing2^−/−^* testes (Fig. 2G). *Ing2^−/−^* testes showed reduced 1N fractions (representing round and elongated spermatids) at 2 months of age, which were almost absent at 6 months. A relative increase in 2N fractions (spermatogonia and somatic cells) in *Ing2^−/−^* testes can be attributed to a failure to complete meiosis II as well as to the presence of somatic cells associated with Leydig cell hyperplasia and Sertoli-cell-only tubules in degenerated seminiferous tubules. An apparent loss of 4N fractions (spermatocytes) in *Ing2^−/−^* testes became evident at 6 months that can be attributed to premature loss of germ cells with ageing. Immunohistochemical (IHC) staining of phosphorylated histone H2AX (γ-H2AX) marked DNA double-strand breaks at leptotene stage associated with meiotic recombination [21], [22] in both *Ing2^+/+^* and *Ing2^−/−^* testes [Fig. 2H-K; black arrows in (I) and (K)]. The γ-H2AX signals were diminished at pachytene stage in *Ing2^+/+^* seminiferous tubules [Fig. 2H,I; white arrows in (I)], as normally expected with the completion of meiotic recombination [21], [22]. In contrast, *Ing2^−/−^* seminiferous tubules were defective in progression to the γ-H2AX-negative pachytene stage (Fig. 2J,K) and instead contained spermatocytes with abnormally accumulated γ-H2AX (Fig. 2K, white arrowheads). These results are consistent with the histopathological observations described above, and indicate a failure to complete meiosis, as well as a progressive loss of germ cells with aging in *Ing2^−/−^* mice.

### Gene expression profiles in *Ing2^−/−^* testes are consistent with spermatogenesis arrest

Gene expression profiling by mRNA microarray analysis using whole testis RNA identified 619 genes differentially expressed (381 downregulated and 238 upregulated, *P*<0.001, FDR<0.04) in *Ing2^−/−^* testes compared with *Ing2^+/+^* testes (GSE18610 at http://www.ncbi.nlm.nih.gov/geo/). *Ing2* was the most downregulated gene (Table S1), confirming the validity of the experimental system. A genelist consisting of genes differentially expressed in *Ing2^−/−^* testes was used to query a database of gene expression profiles during mouse spermatogenesis, including spermatogonia, spermatocytes and spermatids, as well as Sertoli cells, whole seminiferous tubules, and whole testes [GermOnline, http://www.germonline.org [23]]. There was a significant correlation (nonparametric Spearman correlation *P*<0.0001) between gene expression in *Ing2^−/−^* testes and gene expression during mouse spermatogenesis. Specifically, genes differentially expressed in *Ing2^−/−^* testes were positively correlated with genes expressed in Sertoli cells (Spearman r = 0.72 and spermatogonia (Spearman r = 0.70), but negatively correlated with genes expressed in spermatocytes (Spearman r = −0.25) and spermatids (Spearman r = −0.74). These expression profiles further validate the conclusions from our histopathological and IHC findings that indicate failed differentiation of spermatocytes as the major defect caused by Ing2 deficiency.

### Downregulation of genes encoding spermatogenesis-related proteins and chromatin regulatory factors in *Ing2^−/−^* testes

A subset of differentially expressed genes identified by microarray analysis that have well-characterized roles in spermatogenesis and chromatin regulation is listed in Table S1. A group of genes involved in normal differentiation and function of spermatids or sperms, including *Prss21* [24], *Sly* [25], *Spef2* [26], *Ssty2* [27] and the *Speer* family of testis-specific genes [28], were downregulated in *Ing2^−/−^* testes, consistent with impaired progression to these later stages of male germ cell development. Two downregulated genes, *Asb4* (ankyrin repeat and SOCS box-containing protein 4) [29] and *Gzmn* (granzyme N) [30], are believed to be specifically involved in spermatocyte differentiation at pachytene stage, when Ing2 deficiency manifested its effect. The marked downregulation of these two genes in *Ing2^−/−^* testes was validated by qRT-PCR (Fig. S4). It should also be noted that several genes encoding chromatin modifying or associated proteins, including *Setdb2* [31], *Zfp57* [32], *Suv39h2* [12], *Satb2* [33], *Ing3* [34] and *Phf21a* [35], were downregulated in *Ing2^−/−^* testes. In addition to the deregulation of chromatin modifications by loss of Ing2 itself, decreased expression of these proteins may possibly affect chromatin status and contribute to defective spermatogenesis in *Ing2^−/−^* testes. A set of somatic cell-derived transcripts upregulated in testes of HDAC inhibitor-treated mice [14] were not significantly changed in *Ing2^−/−^* testes (Table S2).

### Aberrant chromatin modifications in *Ing2^−/−^* testes

**Figure 3:**
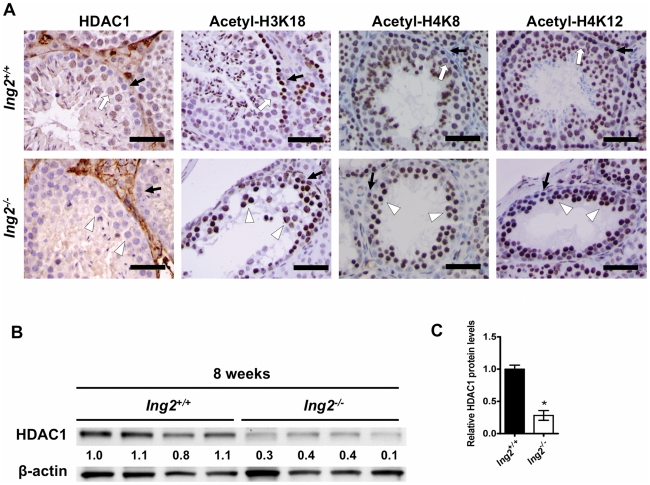
Impaired HDAC1 expression and altered histone acetylation in *Ing2^−/−^* testes. (A) HDAC1, histone H3 acetylated at lysine 18 (acetyl-H3K18), histone H4 acetylated at lysine 8 (acetyl-H4K8) and histone H4 acetylated at lysine 12 (acetyl-H4K12) staining in testes from 8-week-old *Ing2^+/+^* and *Ing2^−/−^* mice. Black arrows indicate spermatogonia and leptotene spermatocytes in both *Ing2^+/+^* and *Ing2^−/−^* testes. White arrows indicate normal pachytene spermatocytes showing HDAC1 induction (leftmost panel) and histone deacetylation (right three panels) in *Ing2^+/+^* testes. White arrowheads indicate abnormal spermatocytes without HDAC1 induction (leftmost panel) and with sustained histone acetylation (right three panels) in *Ing2^−/−^* testes. Scale bars, 50 µm. (B–C) Reduced expression of HDAC1 in *Ing2^−/−^* testes. Whole testis lysates from 8-week-old *Ing2^+/+^* and *Ing2^−/−^* mice (4 mice each) were examined in western blot using anti-HDAC1 antibody. β-actin was a loading control. Relative HDAC1 expression levels (normalized to β-actin) based on quantitative image analysis are shown in (B) and in (C). *P<0.001. Student's *t* test, n = 4 per group. Error bars are s.e.m.

The amounts of H3K4me3, HDAC1 and mSin3A, which all functionally interact with ING2 to regulate chromatin modification [6], [7], were found to be abundant in the testes in *Ing2^+/+^* mice (Fig. S5). In IHC staining, both *Ing2^+/^* ^+^ and *Ing2^−/−^* testes contained HDAC1-negative spermatogonia and leptotene spermatocytes (Fig. 3A, leftmost panels; black arrows). HDAC1 was induced during spermatocyte differentiation into pachytene stage in *Ing2* ^+/+^ testes (Fig. 3A, leftmost panel; white arrow). In contrast, spermatocytes in *Ing2^−/−^* testes showed no significant HDAC1 staining (Fig. 3A, leftmost panel; white arrowheads), consistent with the impaired differentiation to the pachytene stage. Total amounts of HDAC1 were also shown to be decreased in *Ing2^−/−^* testes by Western blot (Fig. 3B,C). Three acetylation sites on core histones (H3K18, H4K8 and H4K12) were acetylated in spermatogonia and leptotene spermatocytes in both *Ing2^+/^* ^+^ and *Ing2^−/−^* testes (Fig. 3A, right three panels each; black arrows). In *Ing2^+/^* ^+^ testes, the deacetylation of these lysine residues was coincident with HDAC1 induction (Fig. 3A, right three panels; white arrows). In contrast, lack of HDAC1 induction in *Ing2^−/−^* seminiferous tubules resulted in accumulated spermatocytes with these sites highly acetylated (Fig. 3A, right three panels; white arrowheads). These results suggest that the meiotic arrest before pachytene stage due to Ing2 deficiency is associated with impaired accumulation of HDAC1 and deregulated histone acetylation.

### p53-dependent and independent apoptosis in *Ing2^−/−^* testes

**Figure 4:**
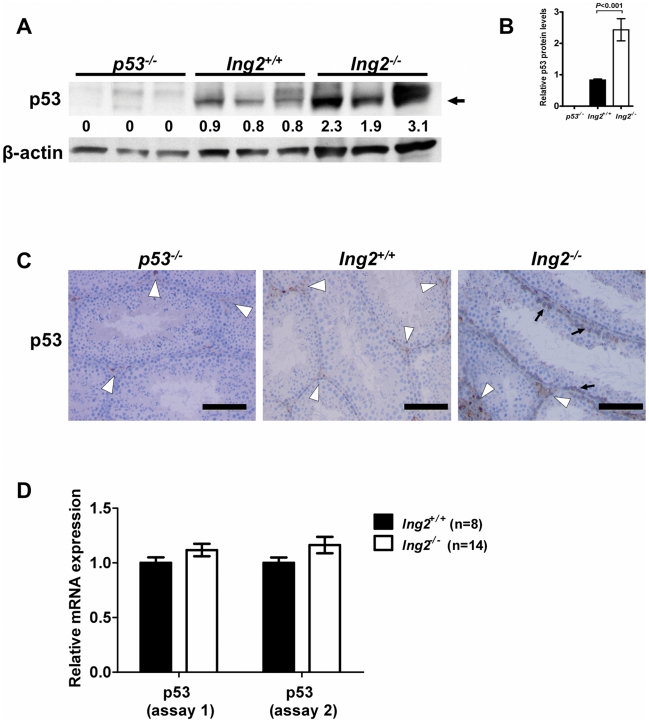
Induction of p53 in *Ing2^−/−^* testes. (A–B) Western blot analysis of p53 protein expression using whole testis lysates from *Ing2^+/+^* and *Ing2^−/−^* testes, as well as *p53^−^* ^/*−*^ testes (negative control), at 8 weeks of age. β-actin was a loading control. Relative p53 expression levels (normalized to β-actin) based on quantitative image analysis are shown in (A) and in (B). Student's *t* test. n = 3 per group. Error bars are s.e.m. (C) p53 staining in testes from 8-week-old *p53^−^* ^/*−*^, *Ing2^+/+^* and *Ing2^−/−^* mice. Black arrows indicate intratubular positive staining in *Ing2^−/−^* testes. Both *Ing2^+/+^* and *Ing2^−/−^* testes, as well as *p53^−^* ^/*−*^ testes to a much lesser extent, show interstitial staining (white arrowheads). Scale bars, 100 µm. (D) Real-time qRT-PCR analysis of p53 mRNA expression. Two independent primers/probe sets (assays 1 and 2) were used. The expression levels in *Ing2^−/−^* testes are shown as the relative values to those in *Ing2^+/+^* testes. Data are mean ± s.e.m. from n = 8 (*Ing2^+/+^*) or n = 14 (*Ing2^−/−^*). No statistically significant difference was observed (Student's *t* test).

Because of the functional association of ING2 with p53 and the well-characterized role of p53 in regulating apoptosis in the testis [36], [37], [38], we next explored the effect of Ing2 deficiency on p53 expression. To this end, 8-week-old *Ing2^+/+^* and *Ing2^−/−^* testes, as well as *p53^−/−^* testes as a negative control, were examined for p53 protein expression. A ∼2.5-fold increase in p53 protein was observed in whole testis lysates of *Ing2^−/−^* mice compared to age-matched *Ing2^+/+^* mice (Fig. 4A,B). Immunohistochemical analysis of p53 protein revealed intratubular staining only in germ cells of *Ing2^−/−^* testes, but not *Ing2^+/+^* or *p53^−/−^* testes (Fig. 4C). No significant increase in p53 mRNA level was found in *Ing2^−/−^* testes (Fig.4D) suggestive of a post-transcriptional mechanism for p53 induction. PUMA, a p53-induced apoptosis effector, was also upregulated in luminal regions of the tubules in *Ing2^−/−^* testes (Fig. S6). These results indicate that endogenous ING2 promotes germ cell survival and differentiation. Loss of ING2 leads to p53 activation *in vivo*, perhaps as an indirect result of testicular degeneration or through a novel regulatory interaction between p53 and ING2.

**Figure 5:**
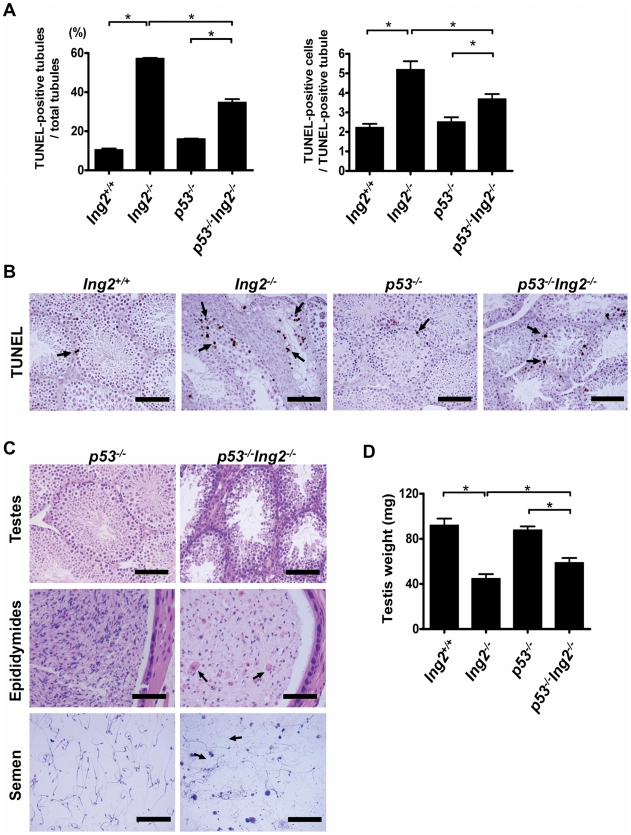
Increased apoptosis and defective spermatogenesis in testes by Ing2 deficiency in the absence of p53. (A, B) TUNEL assay in testes from 8-week-old *Ing2^+/+^*, *Ing2^−/−^*, *p53^−/−^* and *p53^−/−^Ing2^−/−^* mice. (A) The data are shown as ratios of TUNEL-positive tubules to total tubules examined (left) and as average numbers of TUNEL-positive cells per a TUNEL-positive tubule (right). At least 50 tubules were examined in each group. *P<0.001, which corresponds to P<0.05 after applying the stringent Bonferroni correction for 9 multiple comparisons. Student's *t* test, n = 4 per group. Error bars are s.e.m. (B) Representative pictures are shown. Black arrows indicate examples of TUNEL-positive cells. Scale bars, 100 µm. (C) Histological analysis of testes (top, scale bars, 100 µm.), epididymis (middle, scale bars, 50 µm.) and semen (bottom, scale bars, 100 µm.) from 8-week-old *p53^−/−^* and *p53^−/−^Ing2^−/−^* mice. Black arrows indicate degenerated large, round cells in *p53^−^* ^/*−*^ *Ing2^−/−^* epididymis and semen. (D) Average weight of testis from 8-week-old *Ing2^+/+^*, *Ing2^−/−^*, *p53^−/−^* and *p53^−/−^Ing2^−/−^* mice. *P<0.001. Student's *t* test, n = 5 per group. Error bars are s.e.m.

To better characterize the tubular degeneration brought about by Ing2 deficiency, we determined the presence of apoptotic or senescent germ cells in testes at 8 weeks of age. We found that *Ing2^−/−^* testes had significantly increased numbers of TUNEL-positive tubules and TUNEL-positive cells per tubule, compared with those in age-matched *Ing2^+/+^* testes (Fig. 5A, left two bars in both panels). While apoptotic germ cells were observed rarely and close to the basement membrane in *Ing2^+/+^* testes (Fig. 5B, *Ing2^+/+^*), they occurred frequently in spermatocytes in luminal regions of the tubules (Fig. 5B, *Ing2^−/−^*). Neither *Ing2^+/+^* nor *Ing2^−/−^* testes at 8 week of age showed positive staining for senescence-associated β-galactosidase, a marker of cellular senescence [39], in their seminiferous tubules (data not shown).

We next investigated whether disruption of p53 would rescue defective spermatogenesis and enhanced apoptosis of *Ing2^−/−^* mice. Although spermatocytes in *p53^−^* ^/*−*^ males may have an impaired DNA damage response, leading to the formation of a multinucleated giant cell [37], [40], they show otherwise normal spermatogenesis and are fertile [41] (Fig. 5C, *p53^−^* ^/*−*^), allowing us to generate *p53/Ing2* double-knockout mice. Ing2 deficiency in a *p53^−^* ^/*−*^ background reproduced the abnormalities observed in the presence of *p53*, including reduced testis weight (Fig. 5D), degeneration of seminiferous tubules, hypospermia in epididymides, reduced numbers of normal spermatozoa in semen (Fig. 5C, *p53^−^* ^/*−*^ *Ing2^−/−^*) and enhanced apoptosis (Fig. 5A, right two bars in both panels; Fig. 5B, *p53^−/−^* and *p53^−/−^Ing2^−/−^*). However, these abnormalities in *p53^−^* ^/*−*^ *Ing2^−/−^* males were less severe than those in *Ing2^−/−^* males as mentioned above. Quantitative data analysis of the TUNEL assay showed that *p53^−/−^Ing2^−/−^* testes had higher levels of apoptosis than *Ing2^+/+^* and *p53^−/−^* testes but lower levels of apoptosis than *Ing2^−/−^* testes (Fig. 5A), suggesting that Ing2 deficiency induces apoptosis in a p53-dependent manner, which is consistent with p53 induction in *Ing2^−/−^* testes (Fig. 4), as well as in a p53-independent manner. Thus, *p53* deficiency partially rescued the pathological changes due to *Ing2* deficiency. Nevertheless, degenerated large, round cells were accumulated in *p53^−^* ^/*−*^ *Ing2^−/−^* epididymis and semen (Fig. 5C; black arrows), and *p53^−^* ^/*−*^ *Ing2^−/−^* males were still infertile.

### Reduced ING2 expression is associated with impaired spermatogenesis and male infertility in humans

To examine the expression of ING2 in men with low sperm count, morphologically abnormal sperm and/or impaired spermatogenesis, public microarray datasets available at GEO (http://www.ncbi.nlm.nih.gov/geo) and ArrayExpress (http://www.ebi.ac.uk/microarray-as/ae) were queried using the Nextbio search engine (http://www.nextbio.com) (Table 2). Two datasets (GSE6967 and GSE6872) comparing spermatozoa purified from semen samples of infertile teratozoospermic men *versus* normal fertile men [42] showed significantly lower levels of ING2 expression in the former cases. In two independent studies comparing testicular biopsies with impaired spermatogenesis classified according to Johnsen score [43] *versus* full spermatogenesis (GSE4797 and five comparisons in E-TABM-234) [44], [45], decreased expression of ING2 was consistently associated with the spermatogenic defect. Furthermore, ING2 expression was lower in testicular biopsy specimens from non-obstructive azoospermia (NOA) patients than those from obstructive azoospermia (OA) patients (GSE9210) [46], suggesting the functional involvement of reduced expression of ING2 in human male infertility due to defective germ cell development, but not that due to a physical obstruction. Considering that germ cells are the major source of ING2 expression in testis, the finding of low ING2 expression in men with Sertoli-cell only syndrome likely reflects the fact that the spermatogenic cell types are absent from the seminiferous tubules. This association does not *per se* indicate a causative role for ING2 deficiency in human male infertility.

**Table 2:** ING2 expression in spermatogenic pathologies in humans.

Dataset ID (ref)	Specimen	Control group	Test group	Fold change^a^	P value^b^
GSE6967 (42)	Sperm	Normal	Teratozoospermic	−67.4	0.0008
GSE6872 (42)	Sperm	Normal	Teratozoospermic	−3.20	2.4e-5
GSE4797 (44)	Testicular biopsies	Full spermatogenesis	Sertoli-cell-only syndrome^c^	−7.75	0.0112
E-TABM-234 (45)	Testicular biopsies	Full spermatogenesis	Johnsen score 2^d^	−1.52	0.0025
			Johnsen score 3.2^d^	−1.70	0.0006
			Johnsen score 5^d^	−1.62	0.0012
			Johnsen score 7^d^	−1.45	0.0052
			Johnsen score 8^d^	−1.32	0.0267
GSE9210 (46)	Testicular biopsies	Obstructive Azoospermia	Non-obstructive Azoospermia	−1.34	0.0009

a log_2_ ratio (Test group/Control group).

b Welch's *t* test.

c Equivalent to Johnsen score 2.

d Johnsen score 2: no germ cells, Sertoli cells only; Johnsen score 3.2: Sertoli cells and few spermatogonia; Johnsen score 5: no spermatids, many spermatocytes; Johnsen score 7: no late spermatids, many early spermatids; Johnsen score 8: few late spermatids.

### Ing2 is a tumor suppressor gene

To examine whether Ing2 deficiency affects tumorigenesis and the aging processes, we observed *Ing2^+/+^* (n = 22) and *Ing2^−/−^* (n = 28) mice for two years. There was no statistically significant difference in survival between the two genotypes (Fig. S7, *P* = 0.43, Log-rank test). However, detailed histopathological analysis of both groups at time of death showed a significant change in malignant tumor spectrum. The incidence of soft tissue sarcomas was increased in *Ing2^−^* ^/*−*^ mice (*P* = 0.017) (Table 3). The major tumor type observed in *Ing2^−^* ^/*−*^ mice was histiocytic sarcoma (Fig. S8, Table 3), which showed increased incidence preferentially in males for currently unknown reasons (Table S3). Although we confirmed significantly increased frequencies of degenerated seminiferous tubules and oligozoospermic epididymis in *Ing2^−^* ^/*−*^ mice (Tables S4 and S5), pathological examinations of other organs and tissues showed that *Ing2^−^* ^/*−*^ mice had no remarkable difference from *Ing2* ^+/+^ mice in the spectrum of non-malignant lesions, except for increases in benign harderian gland adenomas and in atypical lymphoid hyperplasia of the spleen and a decrease in acinar dilation in prostate (Table S4). *Ing2^−^* ^/*−*^ mice also showed no signs of premature aging, such as hair graying, alopecia, skin atrophy, lordokyphosis, osteoporosis or cataracts. Thus, we currently conclude that Ing2 deficiency by itself does not affect aging phenotypes but enhances spontaneous formation of soft-tissue sarcomas.

**Table 3:** Malignant tumors arising in aging study.

	*Ing2^+/+^ (n* = 22)^a^	*Ing2^−/−^ (n* = 28)^b^	*P* value^c^
**Soft-tissue sarcomas**	3/22 (14%)	13/28 (46%)	**0.017**
Histiocytic sarcoma	1	8	
Hemangiosarcoma	1	2	
Leiomyosarcoma	1	0	
Sarcoma, NOS^d^	0	2	
Neurofibrosarcoma	0	1	
**Lymphomas**	4/22 (18%)	2/28 (7%)	NS
Follicular B-cell lymphoma	2	2	
Splenic marginal zone B-cell lymphoma	2	0	
**Carcinomas**	7/22 (32%)	8/28 (29%)	NS
Lung	6	3	
Duodenum	1	0	
Ovary	0	2	
Liver	0	1	
Pancreas	0	1	
Thyroid	0	1	
**Osteosarcomas**	0/22 (0%)	0/28 (0%)	NS

a 10 female and 12 male.

b 11 female and 17 male.

c Fisher's exact test, NS; Not significant.

d Not otherwise specified.

## Discussion

This study provides evidence that ING2 is an essential regulator of mammalian spermatogenesis by showing that: 1) testes express high levels of ING2 (Fig. 1A,B, Fig. S1); 2) genetic knockout of *Ing2* causes a spermatogenesis defect and male infertility in mice (Figs 1C–G, 2A–F and Table 1); and 3) decreased ING2 expression is highly associated with defective spermatogenesis and male infertility in humans (Table 2). The data indicate that the spermatogenic function of ING2 depends on both its regulatory effect on chromatin (Fig. 3) as well as its functional interaction with p53 (Fig. 4,5).

The spermatocytes in defective *Ing2^−/−^* seminiferous tubules underwent meiotic arrest (Fig. 2G), which was also observed in mouse models deficient for histone methyltransferases [10], [11], [12]. However, unlike these mouse models in which fertility was impaired in both male and female, Ing2 deficiency only affected male fertility, suggesting a distinct role for ING2 in regulating chromatin modification during mammalian germ cell development. Histopathologically, the blocked differentiation of *Ing2^−/−^* spermatocytes into pachytene stage (Fig. 2H–K,5A) most resembled the meiotic defect in testes in mice treated with a HDAC1 inhibitor, trichostatin-A [15]. The ability of ING2 to recruit HDAC1 to H3K4me3 [6], the dynamic regulation of H3K4me3 during zygotene-to-pachytene progression [17], and the accumulation of HDAC1 and the deacetylation of core histones in pachytene spermatocytes in *Ing2* ^+/+^ but not *Ing2^−/−^* mouse testes (Fig. 3A) all suggest that the spermatogenesis defect by Ing2 deficiency is due to a disturbance of the stage-specific histone modifications coordinated by the H3K4me3-ING2-HDAC1 interaction. Our data establish ING2 as a chromatin-associated, non-enzyme protein that is critical to temporal and spatial profiles of chromatin modifications during spermatogenesis. A recent study provides further support to this hypothesis. Suberoylanilide hydroxamic acid (SAHA), an HDAC inhibitor used clinically for the treatment of cancer causes the dissociation of ING2 from the Sin3 deacetylase complex leading to de-repression of downstream genes and growth inhibition[47]. Our findings reveal a plausible mechanism by which HDAC inhibitors may disrupt deacetylase function during spermatogenesis, through disruption of Ing2-bound co-repressor complexes. Consistent with this is also the finding that mice deficient on the Sirt1 protein deacetylase show spermatogenesis defects similar to Ing2-deficient mice [48]. SIRT1 is recruited by ING proteins to negatively regulate mSIN3A/HDAC1 transcriptional repression activity [49]. It is unknown whether an altered expression of one or a few specific genes or a genome-wide change in chromatin status mediates the defects in *Ing2*-deficient mice, as well as in other infertile mice deficient for chromatin modifying factors. The two pachytene-specific genes identified in this study, Asb4 and Gzmn (Fig. S4), deserve further investigation. The somatic cell-derived transcripts differentially expressed in testes of trichostatin-A-treated mice [14] were not significantly affected by Ing2 deficiency (Table S2), in agreement with the findings that germ cells are the major cell type expressing ING2 (Fig. 1B, Fig. S1). *Ing2*-deficient mice should thus be a suitable model to study germ cell-autonomous effects of chromatin modifications on spermatogenesis.

*Ing2^−/−^* mice exhibited phenotypic differences from mice deficient for *Ing1*, the founder member of the ING family, which could be explained by the difference in organ-specific expression profiles [20], the different interacting proteins [1], the different modes of functional interaction with p53 [50], and/or the different p53-independent functions [51]. ING1 expression was low in testes [20] and *Ing1*-deficient mice were fertile [51], [52]. Whereas *Ing1^−^* ^/*−*^ mice had reduced body size [52], *Ing2^−^* ^/*−*^ mice grew normally. Furthermore, while *Ing1^−^* ^/*−*^ mice had elevated incidence of B-cell lymphomas [51], [52], *Ing2^−^* ^/*−*^ mice had elevated incidence of histiocytic sarcoma (Table 3,S3). p19^Arf^-deficient mice exhibit similar phenotypes to *Ing2^−^* ^/*−*^ mice, such as testicular atrophy, increased germ cell apoptosis, loss of sperm and high incidence of histiocytic sarcoma [53], [54]. This most intriguing finding brings up the possibility that ING2 and p19^Arf^, two p53 regulatory proteins, are involved in a common pathway that is aimed to restrict self-renewal and ensure normal differentiation during spermatogenesis as well as prevent tumor development.

p53 regulates apoptosis in testes, which eliminates germ cells with DNA damage induced by irradiation [36], [37]or spontaneously occurring during normal spermatogenesis [38]. p53-independent apoptosis also occurs during spermatogenesis in response to DNA damage and meiotic arrest [37], [55]. Our comparative analysis of apoptosis in *Ing2^+/+^*, *Ing2^−/−^*, *p53^−/−^* and *p53^−/−^Ing2^−/−^* testes (Fig. 5A,B) indicates that both p53-dependent and independent mechanisms of apoptosis were activated by Ing2 deficiency, probably contributing to the elimination of developmentally arrested spermatocytes with unprocessed DNA lesions (Fig. 2J,K). The induction of p53 by Ing2 deficiency (Fig. 4) suggests that a physiological *in vivo* function of endogenous ING2 may be to prevent an illegitimate activation of p53 under non-stressed conditions. However, this functional link between ING2 and p53 is not a sole mechanism by which ING2 ensures normal spermatogenesis and male fertility, as indicated by the mitigated but still significant pathological changes in testes (Fig. 5C,D), the accumulation of abnormal spermatozoa (Fig. 5C) and infertility in *p53^−/−^Ing2^−/−^* males.

Approximately 10% of couples suffer from infertility with approximately equal contributions from men and women [56]. Meiotic arrest during spermatogenesis, as observed in *Ing2^−/−^* mice, is frequently associated with male infertility in humans [57]. However, because of the complexity of the spermatogenic process possibly involving thousands of different genes, a majority of male infertility cases in humans remain not understood [19]. Given a disturbed pattern of chromatin modification in defective spermatogenesis in humans [18] and decreased ING2 expression commonly associated with sperm abnormalities and pathological changes in spermatogenesis in humans (Table 2), we propose that the ING2-mediated chromatin regulation is critical to normal meiotic progression during spermatogenesis in humans as well and may be widely, if not universally, impaired by a number of different spermatogenic defects leading to idiopathic infertility in men. Genetic and epigenetic changes, as well as functional variants, of the *ING2* gene itself also deserve investigation in infertile men.

This study also has significant implications in infertility in men with other diseases, in particular malignant tumors. Patients with testicular cancers are frequently infertile [58], [59], with the degeneration of peritumoral, non-cancerous seminiferous tubules [18], [58]. HDAC inhibitors are under clinical trial in patients with various types of solid and hematologic malignancies [60], [61]. Spermatogenesis is a process highly sensitive to DNA damage by common chemotherapy and radiotherapy [58], [62] and male infertility is a major quality-of-life issue in cancer survivors [58], [63]. We expect that the *Ing2^−/−^* mice generated and characterized in this study can be a model system to study idiopathic and iatrogenic male infertility in humans.

## Materials and Methods

### Generation of *Ing2^−/−^* mice

This mice study was approved by the ACUC, NCI-Frederick and all the guidelines were followed for the study. To construct the targeting vector for *Ing2*, a BAC clone containing the *Ing2* locus was isolated from a 129-mouse genomic library (Genome Systems Inc, St.Louis, MO). A 6.2-kilobase (kb) *Hind*III–*Hind*III fragment encompassing from the first intron to the second intron was cloned into the *Hind*III site of the pBS-SK(-)Pst(-) vector, pBluescript SK(-) (Stratagene, La Jolla, CA) with *Pst*I site removed. A 1.7-kb *Hind*III–*Hind*III fragment within the second intron was cloned into the *Hind*III site of the pLoxpneo vector (a gift from Dr. Chuxia Deng) [64]. A single loxp site was inserted into the *Pst*I site at the first intron within the 6.2-kb fragment cloned in the pBS-SK(-)Pst(-). The resulting plasmid was cleaved with *Not*I and *Xho*I and inserted into the *Not*I and *Xho*I sites of the pLoxpneo containing the 1.7-kb fragment. The final targeting construct was designated pLoxpneoIng2 (Fig. S1a). The targeting construct was linearized and electroporated into ES cells derived from 129/Sv mice. G418-resistant colonies were selected and expanded. The ES clones with correct targeting events were identified by Southern blot and PCR. Heterozygous mice with the targeted allele (*Ing2* ^flox*−*neo/+^) were crossed with each other, resulting in homozygous mice (*Ing2* ^flox*−*neo/flox*−*neo^). These homozygous mice were crossed with EIIa-Cre[65] mice to remove DNA fragments between two loxp sites. This led to the generation of three types of offspring with a deletion of the neomycin resistance cassette (*Ing2* ^+/flox^), the exon 2 (*Ing2* ^+/neo^), or both (*Ing2* ^+/*−*^). The identified *Ing2* ^+/*−*^ mice were then bred with C57BL/6J mice to segregate the EIIa-Cre transgene. *Ing2* ^+/*−*^ mice without EIIa-Cre were mated with each other, leading to the generation of *Ing2* knockout mice (*Ing2^−^* ^/*−*^).

To genotype mice, genomic DNAs were extracted from tails using REDExtract-N-Amp Tissue PCR kit (Sigma, St. Louis, MO) and analyzed by PCR. Thermal cycling was carried out for 35 cycles of denaturation at 94 C° for 30 s, annealing at 55 C° for 30 s and extension at 72 C° for 1 min. The primers were as follows: Ing2-F1 (5′-actgcctcagagcagcaatccca-3′) commonly used for *Ing2* ^+^ and *Ing2^−^* loci, Ing2-R1 (5′-ttgccacatagtcatgaggacc-3′) for amplifying 118-bp product from *Ing2* ^+^ locus, and Ing2-R3 (5′-gatctctgtcacacagtatg-3′) for amplifying 158-bp product from *Ing2^−^* locus.

*p53*-deficient mice were previously described [41]. *p53* and *Ing2* double knockout mice (*p53^−^* ^/*−*^ *Ing2^−^* ^/*−*^) were generated as previously described by Hussain et al [66] for *p53* and *NOS2* double knockout mice.

### Quantitative real-time RT-PCR

Total RNAs were extracted from the whole testes of 8-week-old mice using Trizol (Invitrogen, Carlsbad, CA) according to the manufacturer's protocol. Five micrograms of total RNA were used for the synthesis of first strand cDNA using the SuperScript III First Strand cDNA Synthesis Kit (Invitrogen). To examine ING2 expression in various mouse organs, BD MTC Mouse Panel III was purchased from BD Biosciences (San Jose, CA). Real-time RT-PCR analysis was performed using ABI prism 7900 (Applied Biosystems, Foster City, CA) with Taqman Gene Expression Assays, purchased from Applied Biosystems; Ing2 (Mm00469833_m1), p53 (Mm00441964_g1, assay 1 in this study), p53 (Mm01337166-mH, assay 2 in this study), Asb4 (Mm00480830_m1), and Gzmn (Mm00461850_m1). GAPDH (Applied Biosystems, Mm99999915_g1) was used as internal control. Normalized or relative gene expression was calculated using the equation 2^−ΔCt^ or 2^−ΔΔCt^, respectively, where ΔCt = Ct(Gene)−Ct(GAPDH).

### Gross anatomical and histological examinations

Gross examination of organ weight and morphology on all mice necropsied at 2 weeks to 2 years of age. For routine histopathological examination all tissues were formalin-fixed, embedded in paraffin, sectioned at 5 µm and stained with hematoxylin and eosin (H&E).

### Western blotting

Testes and other organs were removed from 8-week-old mice. Mouse embryo fibroblasts were prepared by standard procedures. For Western blotting, tissues and cells were lysed in RIPA buffer (150 mM NaCl, 1% NP-40, 0.1% sodium deoxycholate, 1 mM EDTA, 0.1% SDS, 10 mM Tris-HCl, pH 7.5) containing complete protease inhibitors (Roche, Indianapolis, IN). Protein samples were run on SDS-polyacrylamide gels (4–20% gradient) (Invitrogen) and blotted onto 0.45-µm nitrocellulose membranes (Bio-Rad Laboratories, Hercules, CA). Membranes were blocked with 5% skim milk in Tris-buffered saline before incubation with primary antibodies: anti-HDAC1 (Millipore, Billerica, MA), anti-H3K4me3 (Abcam, Cambridge, MA), anti-mSin3A (Santa Cruz Biotechnology, Santa Cruz, CA), anti-PUMA (Novus Biologicals Inc), anti-p53 (Santa Cruz Biotechnology, and anti-ING2 [68]. Signals were detected according to standard procedures using ECL detection (Amersham Pharmacia Biotech, Piscataway, NJ). Quantitative image analysis of relative protein expression levels was performed using ImageJ 1.40 g software (http://rsb.info.nih.gov/ij/).

### Immunohistochemical Staining (IHC)

For IHC, testes were fixed in formalin. Antigen retrieval and immunochemical staining were performed as previously described [67] using EnVision System-HRP (DAB) (Dako Cytomation, Carpinteria, CA). Antibodies used for IHC were as follows: anti-AcH3K18 (Cell Signaling Technology, Danvers, MA), anti-AcH4K8 (Cell Signaling Technology), anti-AcH4K12 (Cell Signaling Technology), anti-γ-H2AX (Novus Biologicals Inc, Littleton, CO), anti-HDAC1 (Millipore), anti-p53 (Novocastra, Bannockburn, IL) and anti-ING2 (Sigma).

### TUNEL assay

For terminal deoxynucleotidyltransferase-mediated dUTP-biotin nick end labeling (TUNEL) analysis, formalin-fixed sections were deparaffinized, rehydrated, and pretreated with proteinase K. Apoptotic cells were detected using DeadEnd Colorimetric TUNEL system kit (Promega, Madison, WI) according to the manufacturer's instructions.

### Sperm counts and motility

For each mouse (8-, 16- and 24-week-old) whole epididymis and vas deferens were harvested, cut into 2-mm-long pieces, resuspended in 1 ml of buffer containing 75 mM NaCl, 24 mM EDTA and 0.4% bovine serum albumin. Pieces and sperm fluid were homogenized at 32°C for 10 min to dissociate somatic cells. Sperm cells remaining as a monodispersed suspension were counted on a hemocytometer.

### Testosterone assay

Blood was taken from 8- and 26-week-old male mice (five in each group) that were being housed singly, in separate cages. Female mice were being housed in separate cages of the same room at the time of blood sampling. Serum testosterone level was measured using ELISA kit (Alpha Diagnostic International, San Antonio, TX) according to the manufacturer's instructions.

### Flow cytometry

Testes were excised from 2- and 6-month-old mice (three mice in each group), decapsulated and crushed through 20-gauge needles and 70-µm cell strainers (BD Biosciences) in phosphate-buffered saline. Cells (2×10^6^) were treated with RNase and stained with propidium iodide using a Cycle Test Plus DNA reagent kit (Becton Dickinson, Franklin Lakes, NJ). All fluorescence-activated cell sorting data were analyzed using CELL Quest (version 3.3; Becton Dickinson).

### mRNA microarray analysis

Total RNA samples were isolated from whole testes of *Ing2* ^+/+^ (n = 3) and *Ing2^−^* ^/*−*^ (n = 5) mice at 2-3 months of age using Trizol (Invitrogen). Microarray analysis was performed using Affymetrix platform (Mouse Gene 1.0 ST Array). Data was RMA-normalized using Affymetrix Expression Console, annotated according to NetAffx release: 28 (2009-03-16) and summarized at the gene level. Resulting normalized and annotated probesets were imported into BRB Array Tools (http://linus.nci.nih.gov/BRB-ArrayTools.html). A Class Comparison analysis, based on a Two-sample *T*-test using the random variance model, yielded 1046 probesets differentially expressed between *Ing2^+/+^* and *Ing2^−/−^* testes (*P*<0.001, FDR<0.04). Of those, 300 were not associated with a named gene and were excluded from further analysis. After further filtering for multiple occurrences of a gene ID, 619 unique genes remained, of which 381 were downregulated and 238 were upregulated.

### Bioinformatics analyses

To determine a cell type-specific expression of ING2 in mouse testis, ING2 expression values were extracted from the public Gene Expression Omnibus data repository (GEO, http://www.ncbi.nlm.nih.gov/geo/). A microarray dataset of testes from C57BL/6 mice (accession number GDS409) was interrogated. The expression values for ING2 probesets were identified within the normalized data files available at GEO, and they were subsequently plotted to generate graphical displays.

To examine whether ING2 expression levels are associated with spermatogenic defects and male infertility in humans, we used the datasets available at GEO and ArrayExpress (http://www.ebi.ac.uk/microarray-as/ae), which encompass microarray analysis of testes from men with low sperm count, morphologically abnormal sperm and/or impaired spermatogenesis. Meta-analysis was performed using the Nextbio database of curated and processed high-throughput data (http://www.nextbio.com) by querying for the search term “ING2” and filtering for “male infertility” or “azoospermia”. The analysis output consisted of individual microarray study results that showed differential ING2 expression for the tested comparisons in each dataset. For each microarray study, the fold change and p-value of ING2 for the comparison were indicated.

To compare between the existing datasets of spermatogenesis-associated profiles of gene expression and the differentially expressed genes identified by the mRNA microarray analysis in this study, the gene symbols were compiled into two genesets: upregulated (238) or downregulated (381) in *Ing2^−/−^* testes. These genesets were separately uploaded to GermOnline (http://www.germonline.org [23]), a database of microarray expression profiling data from experiments relevant for the mitotic and meiotic cell cycle, gametogenesis and fertility, from which corresponding expression values were extracted. Expression intensities of 98 and 138 genes were extracted from the upregulated and downregulated genesets, respectively. Expression intensities in Sertoli cells, spermatogonia, spermatocytes, spermatids and tubules were normalized to total testis and plotted separately for each geneset.

### Analysis of spontaneous tumors

Life span and spontaneous tumor incidence were determined in *Ing2^+/+^* and *Ing2^−/−^* mice. Mice with visible tumors and moribund mice showing weight loss or difficulties in moving were sacrificed upon detection. The remaining surviving mice were sacrificed at 24 months of age. Gross anatomical and histological examinations were performed as described above.

### Human subjects

The histology of Mouse testis was compared with human testis obtained from the Armed Forces Institute of Pathology at Water Reed Army Medical Center (AFIP). Use of the human organs was approved by the NIH Office of Human Subjects Research (OHSR) which issued an IRB Exemption (#4545) for the study. Immunohistochemical (IHC) staining of ING2 protein in a normal human testis section was done at the AFIP at Water Reed Army Medical Center.

## Supporting Information

Figure S1 **IHC staining of ING2 in normal human testis.** The rectangular area in (A) is enlarged in the (B). Scale bars are 200 µm in (A) and 100 µm in (B). The specimens in this figure and in Fig.1B were obtained from different individuals. (TIF)

Figure S2 **Generation of** ***Ing2^−/−^*** **mice.** (A) Schematic representation of the targeting vector (pLoxpneoIng2), the wild-type *Ing2* locus and the targeted locus. *Ing2* exon 1, *Ing2* exon 2 and neomycin-resistant gene cassette (TA/neo) are shown as boxes. Red triangles indicate loxP sites. Cleavage sites by *Hind*III, *Not*I, *Xba*I and *Xho*I are also shown. See METHODS for details. (B) DNA genotyping by PCR. (C) Real-time quantitative RT-PCR analysis of ING2 mRNA expression in 8-week-old mouse testes. n = 3 per group. ING2 expression levels (normalized to GAPDH) are shown on a scale of 10^−4^. (D) Western blot analysis of ING2 protein expression in mouse embryo fibroblasts. (TIF)

Figure S3 **Histological analysis of testes from** ***Ing2^+/+^*** **and** ***Ing2*** **^−^** ^***/*−**^ **mice at different ages.** Testes (T) at 2 weeks to 24 months of age, as well as epididymis (E) at 24 months of age, were examined by H&E staining. Scale bars, 200 µm. (TIF)

Figure S4 **Real-time qRT-PCR analysis of Asb4 and Gzmn mRNA expressions.** The expression levels in *Ing2* ^−*/*−^ testes are shown as the relative values to those in *Ing2^+/+^* testes. Data are mean ± s.e.m. from n = 8 (*Ing2^+/+^*) or n = 14 (*Ing2* ^−*/*−^). *P<0.01, **P<0.001, Student's *t* test. (TIF)

Figure S5 **Western blot analysis of H3K4me3, HDAC1 and mSin3A levels in various organs from 8-week-old** ***Ing2^+/+^*** **mice.** β-actin was a loading control. (TIF)

Figure S6 **Immunohistochemical staining of PUMA protein.** Testis sections from 8-week-old *Ing2^+/+^* and *Ing2* ^−*/*−^ mice were used. Spermatogonias with positive PUMA staining [black arrows] were observed in both *Ing2^+/+^* and *Ing2* ^−*/*−^ testes. Abnormal spermatocytes were PUMA-positive in *Ing2* ^−*/*−^ testes (white arrowheads). Scale bars, 100 µm. (TIF)

Figure S7 **Kaplan-Meier survival curves of *Ing2^+/+^* (*n* = 22) and *Ing2*^−^** ^***/*−**^ **(** ***n* = 28) mice.** *P* = 0.43, Log-rank test. (TIF)

Figure S8 **Representative images of histiocytic sarcoma of liver and lung in** ***Ing2*** **^−^** ^***/*−**^ **mice.** Scale bars are 100 µm (left panels) and 50 µm (right panels). (TIF)

Table S1 Selected genes differentially expressed in *Ing2* ^−*/*−^ testes. (DOC)

Table S2 Somatic cell-derived transcripts upregulated by trichostatin-A treatment were not significantly changed in *Ing2* ^−/−^ testes. (DOC)

Table S3 Histiocytic sarcoma arising in aging study. (DOC)

Table S4 Incidence of non-malignant lesions in aging study. (DOC)

Table S5 Degeneration of seminiferous tubules in aging study. (DOC)
