# Supplementary figures and images for: Targeted Disruption of Ing2 Results in Defective Spermatogenesis and Development of Soft-Tissue Sarcomas
Source: PLoS One. 2010 Nov 19;5(11):e15541. doi: 10.1371/journal.pone.0015541 (PMC2988811; doi:10.1371/journal.pone.0015541)

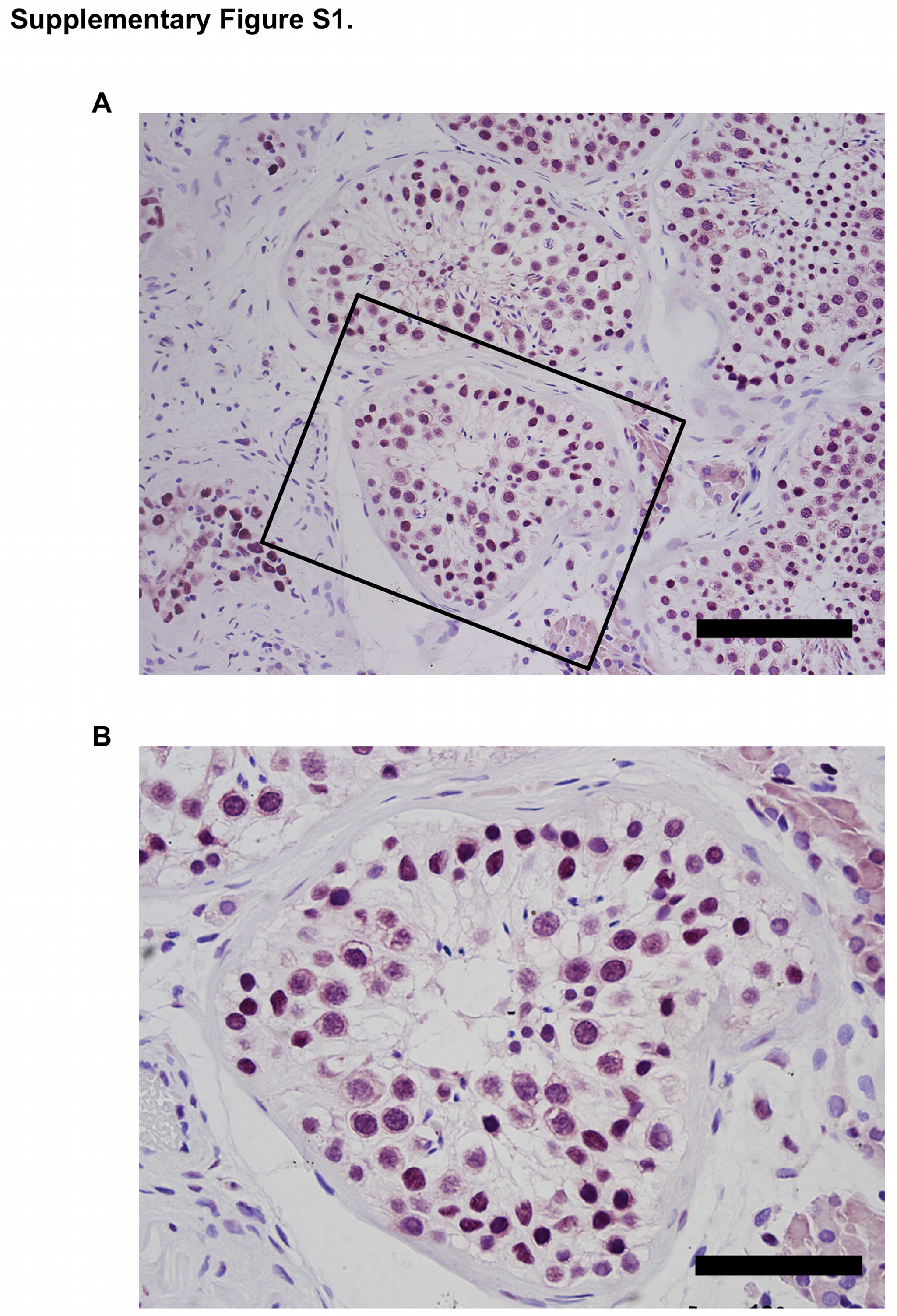

Supplement: Figure S1 — IHC staining of ING2 in normal human testis. The rectangular area in (A) is enlarged in the (B). Scale bars are 200 µm in (A) and 100 µm in (B). The specimens in this figure and in Fig.1B were obtained from different individuals. (TIF) [file pone.0015541.s001.tif]

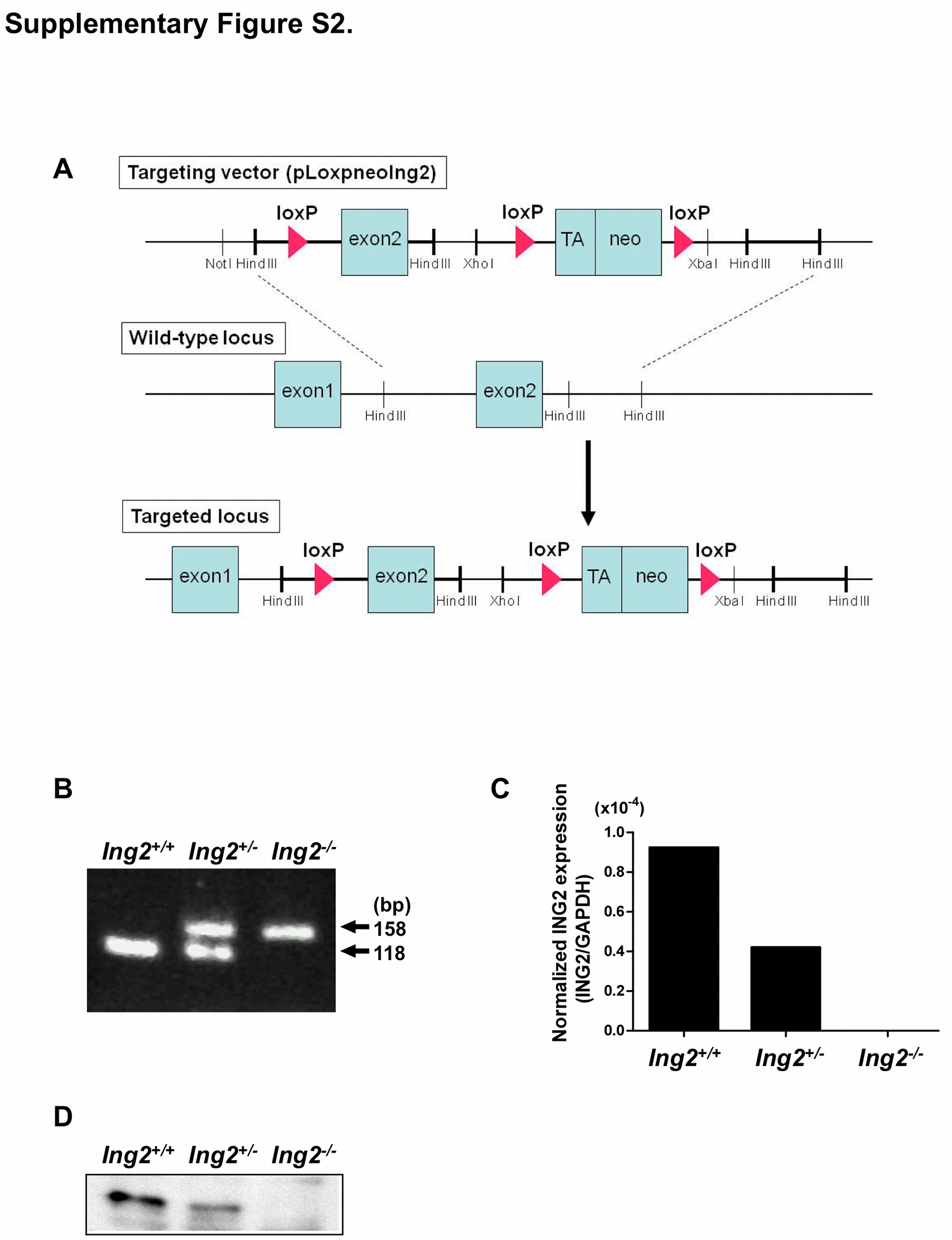

Supplement: Figure S2 — Generation of Ing2−/− mice. (A) Schematic representation of the targeting vector (pLoxpneoIng2), the wild-type Ing2 locus and the targeted locus. Ing2 exon 1, Ing2 exon 2 and neomycin-resistant gene cassette (TA/neo) are shown as boxes. Red triangles indicate loxP sites. Cleavage sites by HindIII, NotI, XbaI and XhoI are also shown. See METHODS for details. (B) DNA genotyping by PCR. (C) Real-time quantitative RT-PCR analysis of ING2 mRNA expression in 8-week-old mouse testes. n = 3 per group. ING2 expression levels (normalized to GAPDH) are shown on a scale of 10−4. (D) Western blot analysis of ING2 protein expression in mouse embryo fibroblasts. (TIF) [file pone.0015541.s002.tif]

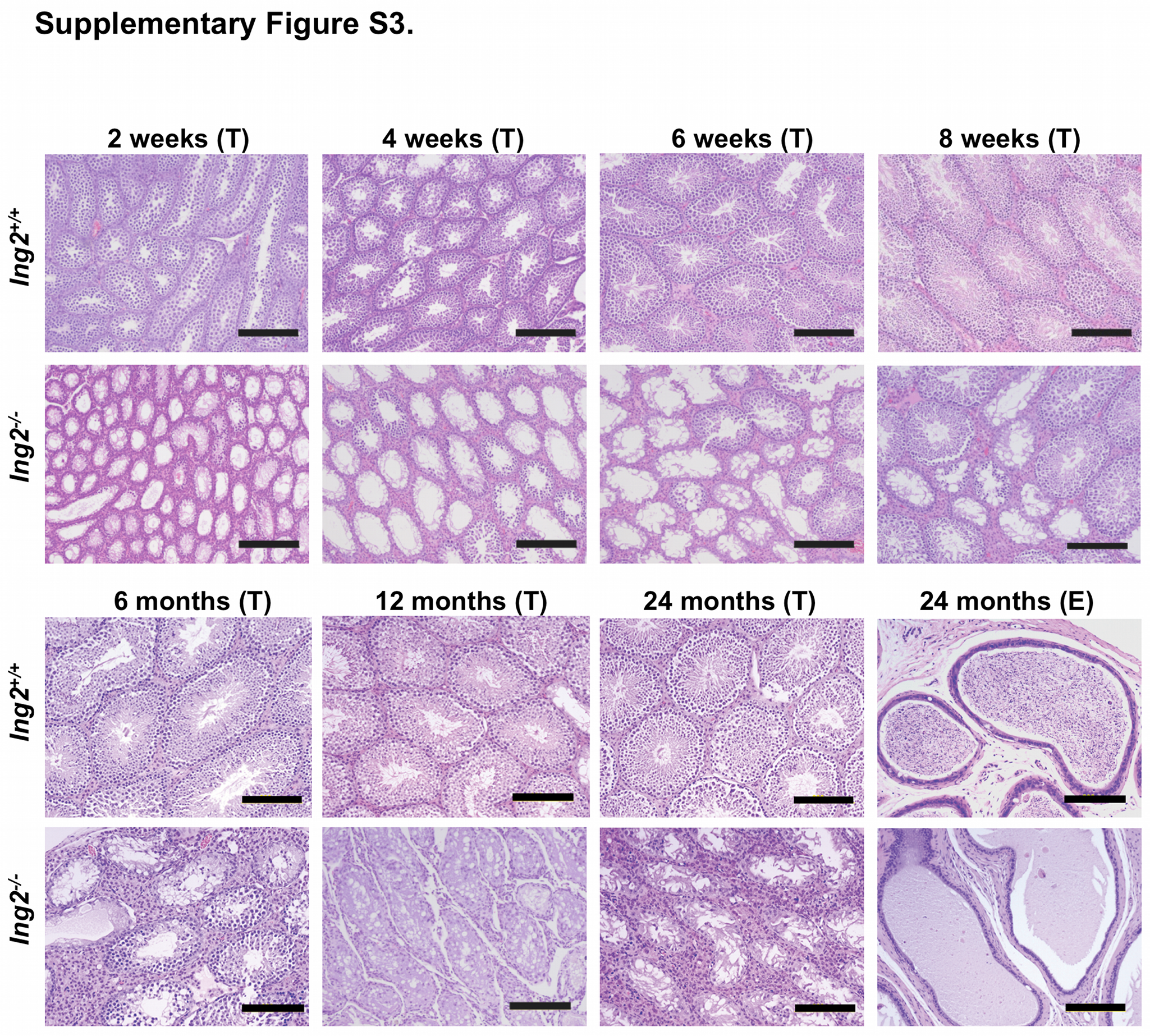

Supplement: Figure S3 — Histological analysis of testes from Ing2+/+ and Ing2 − /− mice at different ages. Testes (T) at 2 weeks to 24 months of age, as well as epididymis (E) at 24 months of age, were examined by H&E staining. Scale bars, 200 µm. (TIF) [file pone.0015541.s003.tif]

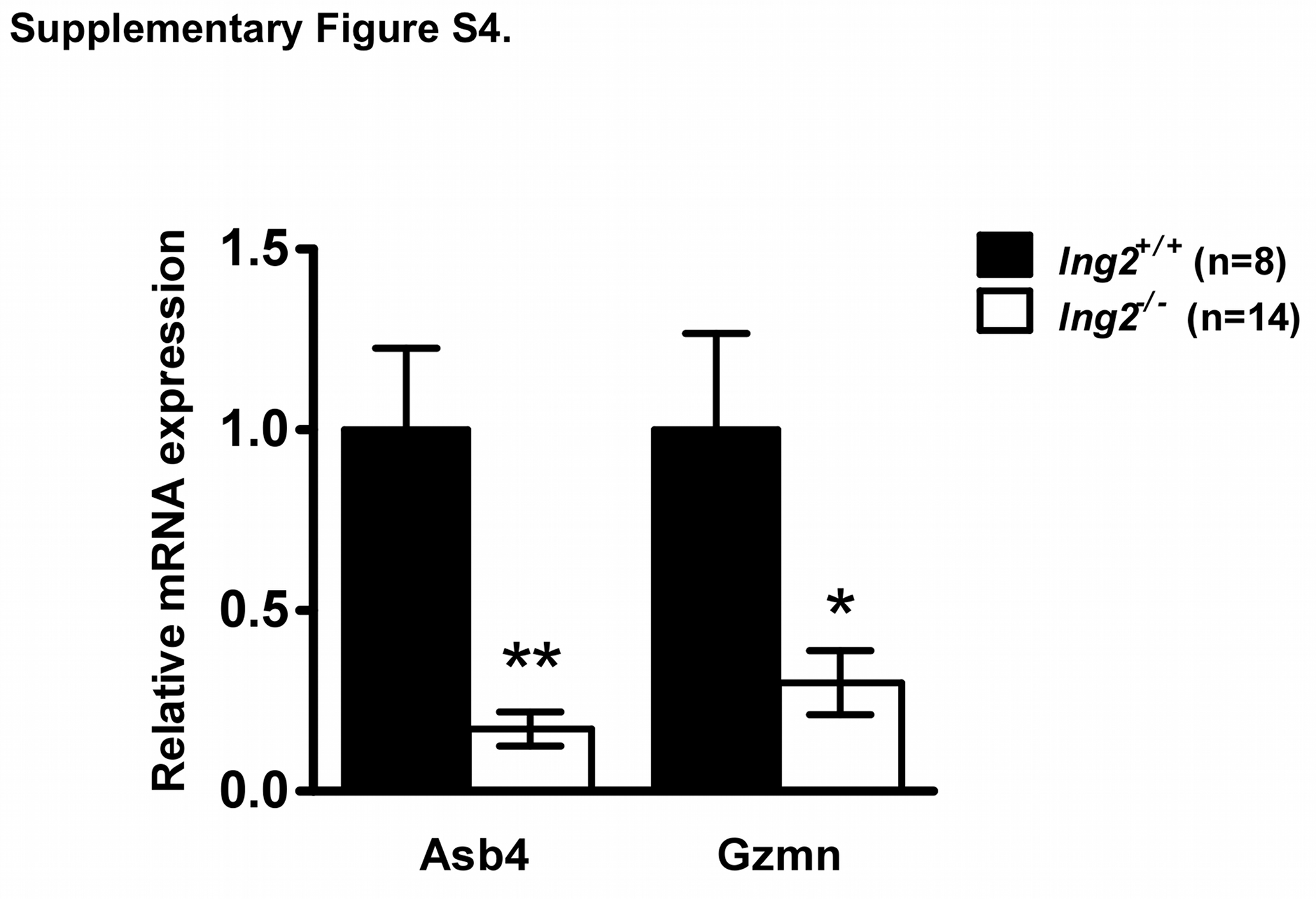

Supplement: Figure S4 — Real-time qRT-PCR analysis of Asb4 and Gzmn mRNA expressions. The expression levels in Ing2 −/− testes are shown as the relative values to those in Ing2+/+ testes. Data are mean ± s.e.m. from n = 8 (Ing2+/+) or n = 14 (Ing2 −/−). *P<0.01, **P<0.001, Student's t test. (TIF) [file pone.0015541.s004.tif]

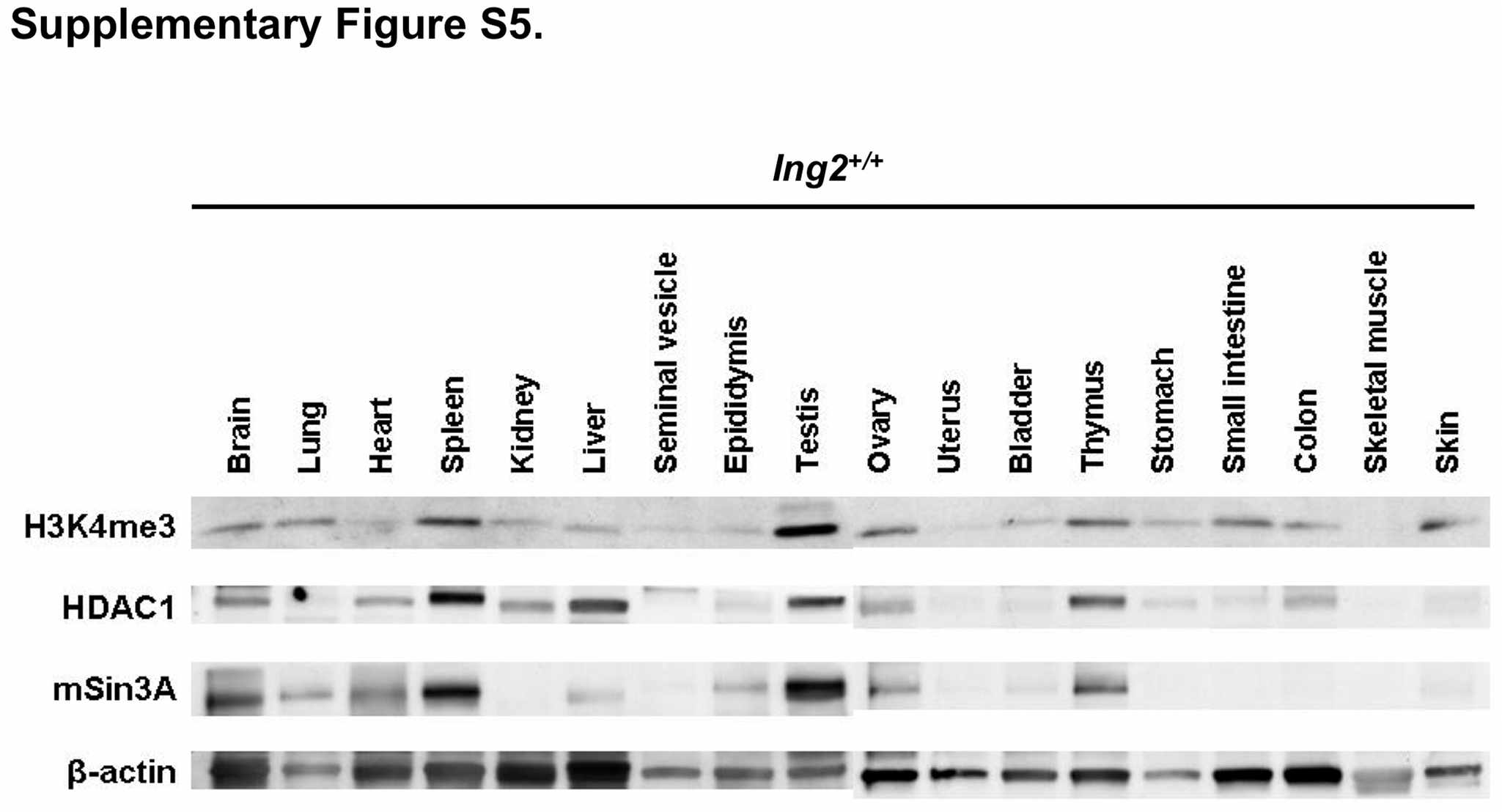

Supplement: Figure S5 — Western blot analysis of H3K4me3, HDAC1 and mSin3A levels in various organs from 8-week-old Ing2+/+ mice. β-actin was a loading control. (TIF) [file pone.0015541.s005.tif]

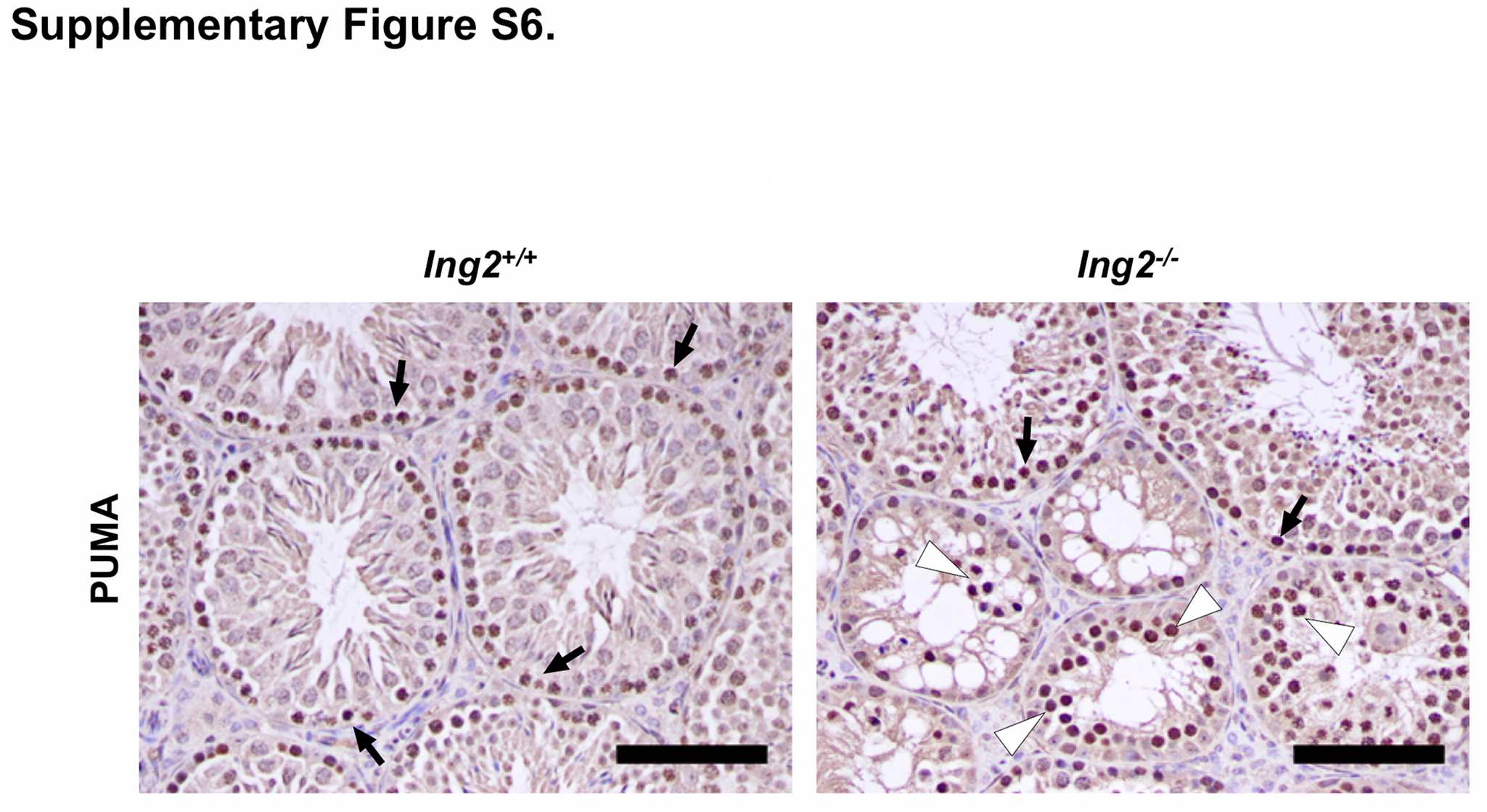

Supplement: Figure S6 — Immunohistochemical staining of PUMA protein. Testis sections from 8-week-old Ing2+/+ and Ing2 −/− mice were used. Spermatogonias with positive PUMA staining [black arrows] were observed in both Ing2+/+ and Ing2 −/− testes. Abnormal spermatocytes were PUMA-positive in Ing2 −/− testes (white arrowheads). Scale bars, 100 µm. (TIF) [file pone.0015541.s006.tif]

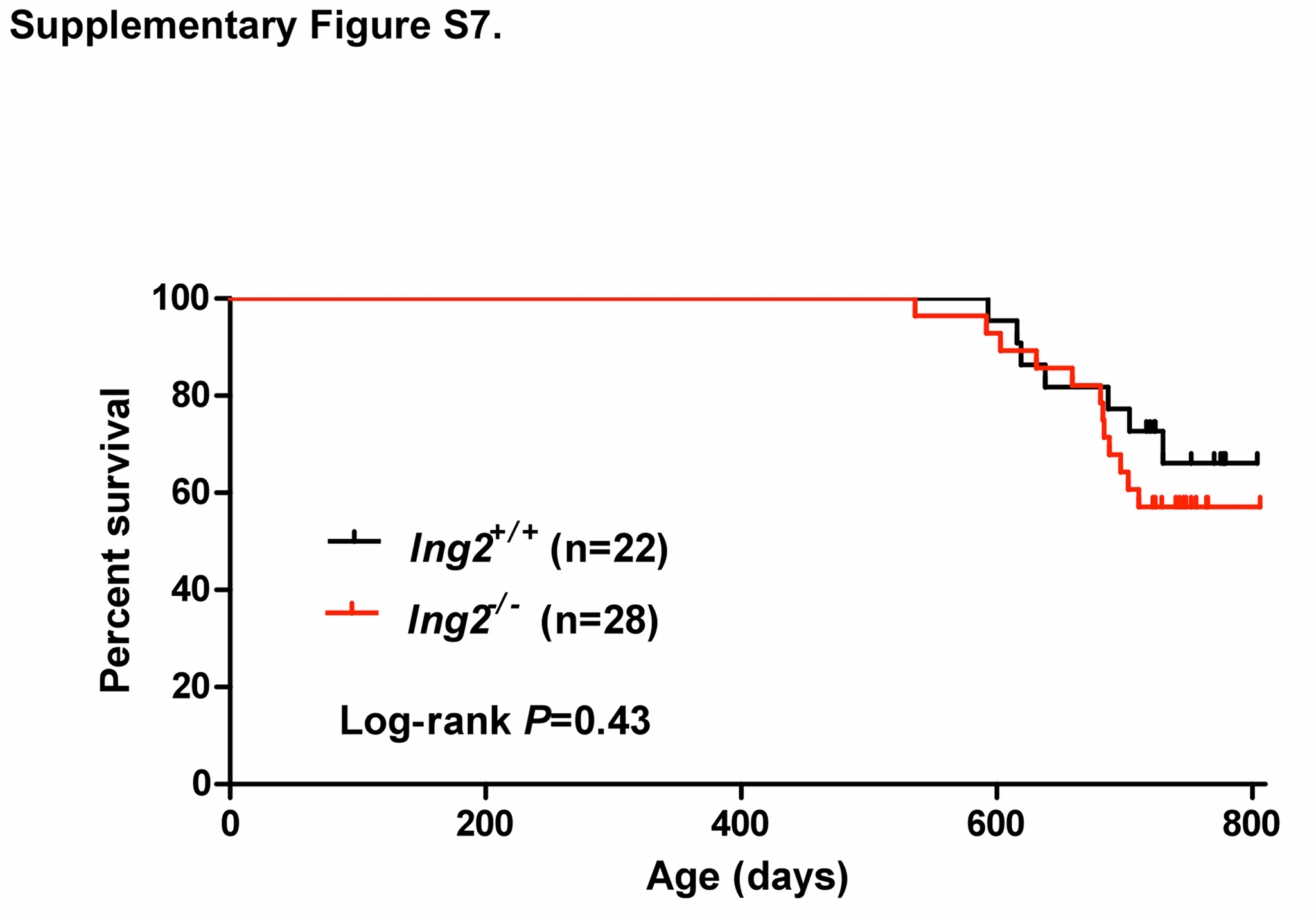

Supplement: Figure S7 — Kaplan-Meier survival curves of Ing2+/+ (n = 22) and Ing2− /− ( n = 28) mice. P = 0.43, Log-rank test. (TIF) [file pone.0015541.s007.tif]

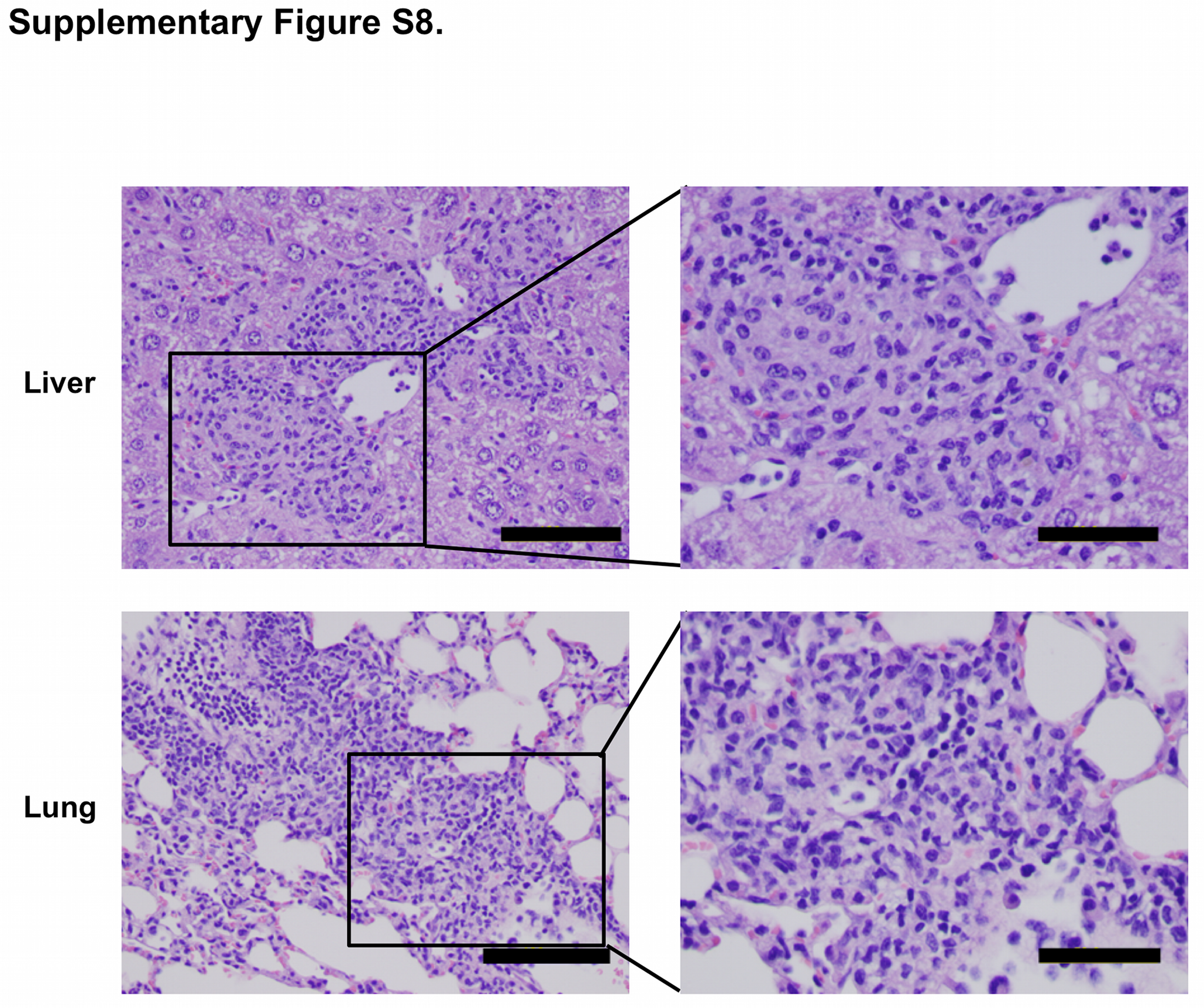

Supplement: Figure S8 — Representative images of histiocytic sarcoma of liver and lung in Ing2 − /− mice. Scale bars are 100 µm (left panels) and 50 µm (right panels). (TIF) [file pone.0015541.s008.tif]
