# Supplementary material for: Targeted Disruption of Ing2 Results in Defective Spermatogenesis and Development of Soft-Tissue Sarcomas
Source: PLoS One. 2010 Nov 19;5(11):e15541. doi: 10.1371/journal.pone.0015541 (PMC2988811; doi:10.1371/journal.pone.0015541)
Supplement: Table S1 — Selected genes differentially expressed in Ing2 −/− testes. (DOC) [file pone.0015541.s009.doc]

**Table S1.** Genes differentially expressed in *Ing2^-/-^* testes (*P*<0.001, FDR<0.04) selected based on their known role in spermatogenesis.

| **Gene ID** | **Gene symbol** | **Gene title** | **Fold change^a^** |
| --- | --- | --- | --- |
| 69260 | Ing2 | inhibitor of growth family, member 2 | **-2.165** |
|  |  |  |  |
| **Spermatocyte differentiation at pachytene stage** | | |  |
| 65255 | Asb4 | ankyrin repeat and SOCS box-containing 4 | **-1.621** |
| 245839 | Gzmn | granzyme N | **-1.228** |
|  |  |  |  |
| **Later stages in spermatogenesis and spermiogenesis** | | |  |
| 57256 | Prss21 | protease, serine, 21 | **-1.009** |
| 624245 | Speer4e | spermatogenesis associated glutamate (E)-rich protein 4e | **-0.957** |
| 382301 | Sly | Sycp3 like Y-linked | **-0.952** |
| 360220 | Speer4d | spermatogenesis associated glutamate (E)-rich protein 4d | **-0.938** |
| 320277 | Spef2 | sperm flagellar 2 | **-0.927** |
| 71026 | Speer3 | spermatogenesis associated glutamate (E)-rich protein 3 | **-0.751** |
| 73526 | Speer4b | spermatogenesis associated glutamate (E)-rich protein 4b | **-0.720** |
| 75657 | Speer4a | spermatogenesis associated glutamate (E)-rich protein 4a | **-0.706** |
| 70009 | Ssty2 | spermiogenesis specific transcript on the Y 2 | **-0.619** |
|  |  |  |  |
| **Chromatin modifying or associated proteins** | | |  |
| 239122 | Setdb2 | SET domain, bifurcated 2 | **-0.766** |
| 22715 | Zfp57 | zinc finger protein 57 | **-0.694** |
| 64707 | Suv39h2 | suppressor of variegation 3-9 homolog 2 | **-0.567** |
| 212712 | Satb2 | special AT-rich sequence binding protein 2 | **-0.438** |
| 71777 | Ing3 | inhibitor of growth family, member 3 | **-0.438** |
| 192285 | Phf21a | PHD finger protein 21A | **-0.409** |
|  |  |  |  |

^a^log_2_ ratio (*Ing2^-/-^* testes / *Ing2^+/+^* testes)*.*

The full list of all differentially expressed genes has been deposited in NCBI's Gene Expression Omnibus (Edgar, et al., *Nucleic Acids Res* 30, 207-210, 2002) and is accessible through GEO Series accession number GSE18610 (http://www.ncbi.nlm.nih.gov/geo/query/acc.cgi?acc=GSE18610).
