# Supplementary material for: Targeted Disruption of Ing2 Results in Defective Spermatogenesis and Development of Soft-Tissue Sarcomas
Source: PLoS One. 2010 Nov 19;5(11):e15541. doi: 10.1371/journal.pone.0015541 (PMC2988811; doi:10.1371/journal.pone.0015541)
Supplement: Table S2 — Somatic cell-derived transcripts upregulated by trichostatin-A treatment were not significantly changed in Ing2 −/− testes. (DOC) [file pone.0015541.s010.doc]

**Table S2.** Somatic cell-derived transcripts upregulated by trichostatin-A treatment^a^ were not significantly changed in *Ing2*^-/-^ testes.

| **Gene symbol** | **Gene title** | **Fold change**^b^ | **Parametric**  ***P* value**^c^ |
| --- | --- | --- | --- |
| Ghrl | ghrelin | -0.485 | 0.0028 |
| Col6a3 | collagen, type VI, alpha 3 | 0.175 | 0.0252 |
| Insl5 | insulin-like 5 | -0.172 | 0.0339 |
| Rln1 | relaxin 1 | 0.116 | 0.0490 |
| Drd4 | dopamine receptor 4 | 0.315 | 0.2158 |
| Lif | leukemia inhibitory factor | 0.098 | 0.2550 |
| Adcyap1r1 | adenylate cyclase activating polypeptide 1 receptor 1 | 0.098 | 0.2735 |
| Mbl2 | mannose-binding lectin (protein C) 2 | -0.080 | 0.4538 |
| Col16a1 | collagen, type XVI, alpha 1 | -0.052 | 0.6816 |
| Irf5 | interferon regulatory factor 5 | 0.003 | 0.8695 |
| Mbl1 | mannose-binding lectin (protein A) 1 | -0.017 | 0.8995 |

^a^Fenic *et al*., *J. Androl.* 29: 172-185, 2008.

^b^log_2_ ratio (*Ing2^-/-^* testes / *Ing2^+/+^* testes)*.*

^c^Genes are sorted by *P* value of the univariate test. *P*≥0.001, not significant.
