# Supplementary material for: Targeted Disruption of Ing2 Results in Defective Spermatogenesis and Development of Soft-Tissue Sarcomas
Source: PLoS One. 2010 Nov 19;5(11):e15541. doi: 10.1371/journal.pone.0015541 (PMC2988811; doi:10.1371/journal.pone.0015541)
Supplement: Table S3 — Histiocytic sarcoma arising in aging study. (DOC) [file pone.0015541.s011.doc]

**Table S3.** Histiocytic sarcoma arising in aging study.

|  |  | ***Ing2^+/+^*** | ***Ing2^-/-^*** | ***P* value^a^** |
| --- | --- | --- | --- | --- |
|  |  |  |  |  |
| **Histiocytic sarcoma** | **Male** | 0/12 | 6/17 (35.3%) | **0.028** |
|  |  |  |  |  |
|  | **Female** | 1/10 (10%) | 2/11 (18%) | 1.000 |
|  |  |  |  |  |
|  | **Total** | 1/22 (5%) | 8/28 (29%) | 0.060 |
|  |  |  |  |  |

^a^Fisher’s exact test.
