# Supplementary material for: Targeted Disruption of Ing2 Results in Defective Spermatogenesis and Development of Soft-Tissue Sarcomas
Source: PLoS One. 2010 Nov 19;5(11):e15541. doi: 10.1371/journal.pone.0015541 (PMC2988811; doi:10.1371/journal.pone.0015541)
Supplement: Table S4 — Incidence of non-malignant lesions in aging study. (DOC) [file pone.0015541.s012.doc]

**Table S4.** Incidence of non-malignant lesions in aging study.

| **Organ** | **Pathology** | ***Ing2^+/+^***  ***(n*=22)^a^** | ***Ing2^-/-^***  *(****n*=28)^b^** |
| --- | --- | --- | --- |
| | Adrenal | | --- | |  | |  | | Brain | |  | | Duodenum  Epididymis | |  | | Eye | |  | |  | |  | | Femur | |  | | Gall bladder | |  | | Harderian gland  Heart | |  | | Kidney | |  | |  | |  | | Liver | |  | |  | |  | |  | |  | | Lymph node, mesenteric  Lung | |  | |  | |  | |  | | Mammary gland | |  | | Nose | |  | | Ovary | |  | |  | | Pancreas | |  | |  | |  | | Parathyroid | |  | | Pituitary | |  | |  | |  | | Prostate | |  | | Salivary gland | | Seminal vesicles | |  | | Skin | |  | | Spinal cord | |  | | Spleen | |  | |  | |  | |  | |  | | Stomach | |  | |  | |  | | Testis | |  | |  | | Thyroid | |  | |  | | Tongue | |  | | Urinary bladder | |  | |  | | Uterus | |  | |  | | Vertebra | |  | | | Hyperplasia, subcapsularr cell | | --- | | Lipogenic pigment | |  | | Mineralization | |  | | Adenoma  Oligozoospermia | |  | | Cataract | | Keratitis | | Degeneration, retina | |  | | Arthropathy, stifle | |  | | Cyst, intramural | |  | | Adenoma  Cardiomyopathy | |  | | Cysts | | Glomerulonephropathy | | Hydronephrosis | |  | | Adenoma  Cirrhosis | | Hapatitis | | Inflammation, subacute | | Necrosis | | Vacuolation, hepatocellular | |  | | Hemangioma  Adenoma  Hemorrhage | | Lymphocytic infiltrate | | Pneumocystis carinii | | Pneumonia | |  | | Dilation, duct | | Hyperplasia | |  | | Hyalinosis | |  | | Adenoma, tubulostromal  Atrophy | | Cyst | | Hyperplasia | | Atrophy | | Hyperplasia, islet cell | | Lymphocytic infiltrate | |  | | Cyst, embryonic rest | |  | | Adenoma  Cyst | | Hyperplasia, pars distalis | | Hyperplasia, pars intermedia | |  | | Dilation, acinar | |  | | Arteritis | | Lymphocytic infiltrate | |  | | Dilatation, secretory | | Hemorrhage | |  | | Ulcer | |  | | Spongiosis/Vacuolation | |  | | Angiectasis | | Arteritis | | Extramedullary hematopoiesis | | Hyperplasia, lymphoid | | Hyperplasia, lymphoid, atypical | |  | | Dilatation, gland | | Hyperplasia | | Inflammation | |  | | Degeneration seminiferous tubules | | Hemorrhage | |  | | Adenoma  Cyst, embryonic rest | | Dilatation, follicle | |  | | Arteritis | |  | | Arteritis | | Dilatation | | Lymphocytic infiltrate | |  | | Fibrosis | | Hyperplasia, cystic endometrial | |  | | Hyperplasia granulocytic | | | 14/22 (64%) | | --- | | 7/22 (32%) | |  | | 8/22 (36%) | |  | | 0/22 (0%)  1/12 (8.3%) | |  | | 0/20 (0%) | | 3/20 (20%) | | 1/20 (5%) | |  | | 2/22 (9%) | |  | | 1/22 (5%) | |  | | 0/22 (0%)  3/22 (14%) | |  | | 5/22 (23%) | | 18/22 (82%) | | 0/22 (0%) | |  | | 3/22 (14%)  1/22 (5%) | | 1/22 (5%) | | 13/22 (59%) | | 0/22 (0%) | | 5/22 (23%) | |  | | 1/22 (5%)  3/22 (14%)  1/22 (5%) | | 19/22 (86%) | | 0/22 (0%) | | 3/22 (14%) | |  | | 1/10 (10%) | | 0/10 (0%) | |  | | 14/22 (64%) | |  | | 0/10 (0%)  4/10 (40%) | | 2/10 (20%) | | 0/10 (0%) | | 0/22 (0%) | | 18/22 (82%) | | 4/22 (18%) | |  | | 0/19 (0%) | |  | | 1/20 (5%)  1/20 (5%) | | 3/20 (15%) | | 6/20 (30%) | |  | | 8/12 (67%) | |  | | 1/22 (5%) | | 20/22 (91%) | |  | | 9/12 (75%) | | 1/12 (8%) | |  | | 0/22 (0%) | |  | | 0/22 (0%) | |  | | 1/22 (5%) | | 1/22 (5%) | | 5/22 (23%) | | 2/22 (9%) | | 0/22 (0%) | |  | | 0/22 (0%) | | 4/22 (18%) | | 1/22 (5%) | |  | | 1/12 (8.3%) | | 0/12 (0%) | |  | | 0/22 (0%)  0/22 (0%) | | 3/22 (14%) | |  | | 1/22 (5%) | |  | | 1/22 (5%) | | 0/22 (0%) | | 10/22 (45%) | |  | | 1/10 (10%) | | 5/10 (50%) | |  | | 2/22 (9%) | | | 11/28 (39%) | | --- | | 12/28 (43%) | |  | | 7/28 (25%) | |  | | 1/28 (4%)  17/17 (100%)** | |  | | 2/28 (7%) | | 5/28 (18%) | | 2/28 (7%) | |  | | 6/28 (21%) | |  | | 0/28 (0%) | |  | | 6/28 (21%)*  5/28 (18%) | |  | | 3/28 (11%) | | 23/28(82%) | | 1/28 (4%) | |  | | 4/28 (14%)  0/28 (0%) | | 1/28 (4%) | | 15/28 (54%) | | 3/28 (11%) | | 4/28 (14%) | |  | | 0/28 (0%)  1/28 (4%)  0/28 (0%) | | 25/28 (89%) | | 1/28 (4%) | | 6/28 (21%) | |  | | 0/10 (0%) | | 2/10(20%) | |  | | 17/28 (61%) | |  | | 1/11 (9%)  6/11 (55%) | | 1/11 (9%) | | 2/11 (18%) | | 1/28 (4%) | | 18/28 (64%) | | 2/28 (7%) | |  | | 1/26 (4%) | |  | | 2/26 (8%)  2/26 (8%) | | 0/26 (0%) | | 7/26 (35%) | |  | | 3/15 (20%)* | |  | | 1/28 (4%) | | 20/28 (71%) | |  | | 9/17 (53%) | | 1/17 (6%) | |  | | 1/27 (4%) | |  | | 2/28 (7%) | |  | | 0/28 (0%) | | 0/28 (0%) | | 6/28 (21%) | | 1/28 (4%) | | 7/28 (25%)* | |  | | 2/28 (7%) | | 5/28 (18%) | | 0/24 (0%) | |  | | 17/17 (100%)** | | 1/17 (6%) | |  | | 1/28 (4%)  2/28 (7%) | | 9/28 (32%) | |  | | 0/28 (0%) | |  | | 0/28 (0%) | | 2/28 (7%) | | 9/28 (32%) | |  | | 0/11 (0%) | | 8/11 (73%) | |  | | 0/24 (0%) | |

^a^10 female and 12 male.

^b^11 female and 17 male.

**P*<0.05, ***P*<0.001, Fisher’s exact test.
