# Supplementary material for: Targeted Disruption of Ing2 Results in Defective Spermatogenesis and Development of Soft-Tissue Sarcomas
Source: PLoS One. 2010 Nov 19;5(11):e15541. doi: 10.1371/journal.pone.0015541 (PMC2988811; doi:10.1371/journal.pone.0015541)
Supplement: Table S5 — Degeneration of seminiferous tubules in aging study. (DOC) [file pone.0015541.s013.doc]

**Table S5.** Degeneration of seminiferous tubules in aging study.

| **Grade** | ***Ing2^+/+^***  ***(n*=12)** | ***Ing2^-/-^***  ***(n*=17)** |
| --- | --- | --- |
| 1(+), Minimal   | 2(+), Mild | | --- | | 3(+), Moderate | | 4(+), Severe |   Total | | 0/12 (0%) | | --- | | 0/12 (0%) | | 1/12 (8%) | | 0/12 (0%) |   1/12 (8%) | | 0/17 (0%) | | --- | | 0/17 (0%) | | 4/17 (24%) | | 13/17 (76%)** |   17/17 (100%)** |

***P*<0.001, Fisher’s exact test.
